# Self-Assembled Materials Incorporating Functional Porphyrins and Carbon Nanoplatforms as Building Blocks for Photovoltaic Energy Applications

**DOI:** 10.3389/fchem.2021.727574

**Published:** 2021-10-01

**Authors:** Boyang Mao, Benjamin Hodges, Craig Franklin, David G. Calatayud, Sofia I. Pascu

**Affiliations:** ^1^ Department of Chemistry, University of Bath, Bath, United Kingdom; ^2^ Cambridge Graphene Centre, Engineering Department, University of Cambridge, Cambridge, United Kingdom; ^3^ Centre for Sustainable and Circular Technologies (CSCT), University of Bath, Bath, United Kingdom; ^4^ Department of Electroceramics, Instituto de Ceramica y Vidrio (CSIC), Madrid, Spain

**Keywords:** supramolecular chemistry, functional graphene materials, porphyrins, donor/acceptor interactions, net zero, sustainable processes

## Abstract

As a primary goal, this review highlights the role of supramolecular interactions in the assembly of new sustainable materials incorporating functional porphyrins and carbon nanoplatforms as building blocks for photovoltaics advancements.

## Sustainable Energy Production in Current Socioeconomic Context

Production and consumption of energy is a basic element in daily life and crucial to societal development. The demand for energy is increased continuously with the evolution of civilisations. The increase of the human population, urbanisation, modernisation, and technology development all go along with the increased demand for energy supply. It is predicted that the growth in global energy demand will rise harshly over the coming year rendering the current reliance on fossil fuels, which is a bottleneck for continuous economic growth worldwide. Currently, the world heavily relies on fossil fuels to meet the vast majority of its energy needs. Fossil fuels, for instance, oil, gas, and coal, which were generated by natural processes such as anaerobic decomposition of buried, dead organisms over million years, are providing almost 80% of the global energy demands (Asif and Muneer, 2007; World Energy Outlook, 2020 – Analysis – IEA). The fact that humanity faces is that a significant amount of energy currently being consumed across the world has adverse implications on the environment of the Planet, and this triggered the current race to “net zero.” The dominant purveyors of global energy, fossil fuels, are seen as placing merciless effects on ecosystems. The significant consumption of fossil fuels leads to the dramatic emission of greenhouse gas (CO_2_, CH_4_, etc.), which impact directly the environment and contribute to climate change. It has been reported that 160,000 people die each year due to the side effects of climate change, which includes malnutrition, malaria, and spread of epidemic diseases, which follow in the wake of floods, droughts, and warmer Earth temperatures, and the numbers of victims may double by the end of 2020, according to the World Health Organization (2018) (Climate change and health; Asif and Muneer, 2007).

It has been well known that the amount of fossil fuel generated by the current sources decrease and, by facing the difficult climate challenges, the current energy system is unable to cope with future energy demands. The production and consumption of nuclear energy, which is mainly employed in developed counties, together with fossil fuels are strictly linked to increased threats to biological diversity, environmental degradation that affects human health and quality of life and affects ecological balance. Therefore, if the rapidly increasing global energy needs are to be met without irrevocable environmental damage, there should have to be a worldwide drive to create and exploit energy systems that are not detrimental to the life of current and future generations and do not exceed the current carrying capacity of ecosystems. This situation has become even more crucial in recent years, the variability in the price of crude oil (World Energy Outlook, 2020 – Analysis – IEA), considerations about natural gas fracking and the economic crisis, make people less concerned about achieving sustainable energy development that does not compromise the future.

Sustainable energy sources, which are naturally replenished, inexhaustible, and widely available on a human timescale such as sunlight, wind, rain, tides, and geothermal heat, have the potential to provide energy services with almost nil emissions of both air pollutants and greenhouse gases. Sustainable energy can replace fossil fuels in four distinct areas: electricity generation, hot water/space heating, motor fuels, and rural energy services. Currently, sustainable energy is only contributing to 13.5% (Asif and Muneer, 2007; Department for Business, 2020; Renewables – Global Energy Review, 2020 – Analysis – IEA; Report extract Renewables, 2020; World Energy Outlook, 2020 – Analysis – IEA) of the total energy needs. Sustainable energy resources have the capacity to meet the current and future energy requirements of the world and minimise the reliance on fossil or nuclear fuels.

The development and use of sustainable energy sources have significant benefits including the enhancement of diversity in energy supply markets, help reduce local and global environmental impacts, provide commercially alternative options to meet specific energy service demands, and contribute to securing long-term sustainable energy supplies and also creating new employment opportunities practically in developing country and rural area. It is also noticed that the cost of energy generated from these renewable resources is significantly reduced with the development of high technology.

Over the last two decades, solar and wind energy systems have experienced rapid growth and dominated the sustainable energy market (Chu and Majumdar, 2012; Department for Business, 2020; Renewables – Global Energy Review, 2020 – Analysis – IEA; Report extract Renewables, 2020; World Energy Outlook, 2020 – Analysis – IEA). This rapid growth was contributed by several reasons, like the decreasing capital cost and continued improvement in performance characterisation. Compared with wind energy, solar energy is still being investigated and has huge potential in the technology revolution. The economic and policy mechanisms for developing solar energy support the widespread dissemination and rapid evolution. Chemists, material scientists, and other physical scientists have started the race for high-performance materials of relevance to sustainable energy applications.

Therefore, the topic of sustainable energy production raised to prominence in both academic research and governmental policy over the past 20 years. The diminishing supplies of oil, increased population, and the increase in the demand for energy mean that alternative methods for the production of energy are greatly desired. This is in line with global developments whereby renewable electricity generation rose by nearly 5% in 2020 (notwithstanding the current COVID-related challenges that caused supply chain disruptions that have paused or delayed economic activities in several key regions) and is expected to continue to raise at this rate globally throughout to the end of 2021 (Renewables – Global Energy Review, 2020 – Analysis – IEA). Within the United Kingdom, the total energy consumption in 2019 (pre-COVID crisis) raised to 3,600 kWh per annum for electricity and 13,600 kWh per annum for gas (Department for Business, 2020).

**FIGURE 1:**
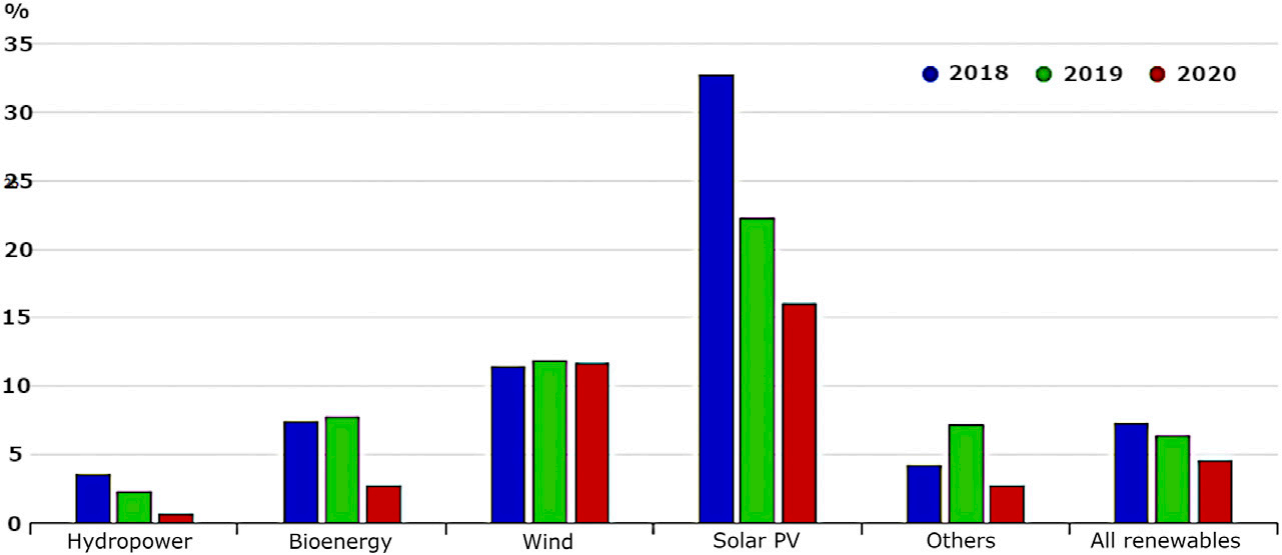
Annual growth for renewable electricity generation by source.

Although overall the demand for electricity has generally been declining since 2010 due to the introduction of energy-saving systems throughout the country, the domestic production of energy is rapidly falling as production and consumption of primary energy sources such as coal and gas are reduced for both financial reasons and the meeting of carbon emission targets. This means that energy produced from sustainable sources such as wind, hydro, and solar energy is being employed to make up the difference, increasing at a steady rate since 1990. Overall, in the United Kingdom, the renewable energy sources have grown at an average annual rate of 2.0%, which is slightly higher than the growth rate of world TES, 1.8%. Growth has been especially high for solar PV and wind power, which grew at average annual rates of 36.5 and 23.0%, respectively Figure 1). Interestingly, a marked increase was seen from 2012 to 2013 when the energy produced from sustainable sources in the United Kingdom grew by 30% to contribute to a total of 14.9% overall of the electricity produced in the United Kingdom at that date and continue to make a significant contribution to the energy consumption.

Current solar cell technologies rely on materials and processes that require a large input of energy and thus increase their overall CO_2_ equivalence. Studies have shown that PV systems based on silicon produce 50–250 g-CO_2_/kWh over their lifetime which is higher than most other renewable sources of electricity (although still lower than conventional coal, oil, or gas systems) (Renewables – Global Energy Review, 2020 – Analysis – IEA; Report extract Renewables, 2020; World Energy Outlook, 2020 – Analysis – IEA). Producing low-cost and low-impact solar cells is the key to the continuation of this rising technology.

Industrially, several different classes of devices and underlining materials are being produced for applications in sustainable technologies. These include (but are not limited to) the organic electronic areas of relevance to organic photovoltaics, organic photodiodes, OLEDs, and thin-film transistors development. Using organic semiconductors as the basis of device manufacturing in sustainable technologies is widely acknowledged due to the low-cost manufacturing methods, based on evaporating or printing on a variety of substrates.

On the other hand, direct storage of solar energy is very difficult and even unworkable outside research environments; in this sense, solar energy is usually first converted into other forms of energy and then stored (Badenhorst, 2019; Brunetti et al., 2019). The most commonly used methods currently are the conversion of energy into thermal energy (Dahash et al., 2019), conversion into biomass energy (Bentsen and Møller, 2017), conversion into chemical energy (Wang et al., 2020), and conversion and storage of solar energy electrochemically (Meng et al., 2019; Wang et al., 2021a), being the last one which offers the best practical prospects. To realise the potential of solar-to-electrochemical energy conversion and storage, the integration of solar cells with electrochemical energy storage (EES) devices is necessary (Gurung and Qiao, 2018). Specifically, this involves a solar cell as the energy harvesting unit and an EES device [e.g., a rechargeable battery or supercapacitor (SC)] as the energy storage unit, resulting in what is known as a “photo-charging” process. Thus, the involvement of innovative EES units is as important as solar cells. Like in the case of solar cells, carbon-based functional materials can also improve the performance of rechargeable batteries and SCs (Ren et al., 2020). As is well known, carbon black can greatly increase the conductivity of electrode materials, thus bringing better charge/discharge characteristics to batteries. Carbon coatings on the surface of electrode materials can also improve the cyclic stability of EES units in integrated devices. For example, the involvement of novel materials such as carbon nanotubes (CNTs) and graphene in the design elements can be considered, by directly using these as active electrode materials for batteries and SCs, due to their high electronic conduction, large surface area, and tuneable electrochemical activity (Li et al., 2019; Wang et al., 2021b). Apart from the aforementioned functions, nanocarbon arrays (e.g., CNT- and graphene-based fibres and membranes) can act as ideal flexible substrate materials for integrated devices to achieve flexibility, portability, and wearability (Lu et al., 2019; Guo et al., 2021).

Overall, it is clear that carbon materials and their analogues play very critical roles in the configuration design and performance enhancement of integrated devices of high relevance to sustainable technology applications (Shi et al., 2019). However, due to the breadth of the topic, the authors will focus in this review on the energy harvesting component and particularly on aspects of molecular designs involved in innovative solar cells technologies and related applications.

## Overview on Solar Cell Technologies

The sun provides 3 × 10^15^ GJ a year or about 10,000 times more than the global population currently consumes. This would easily satisfy our needs, provided a large enough proportion of the Earth was covered with solar cells (0.1% of the Earth’s surface with solar cells with an efficiency of 10%) (Grätzel, 2005). The discovery of the ability to convert solar energy into electricity was originally published by Becquerel who noted a photocurrent when platinum electrodes, covered with silver bromide or silver chloride, were illuminated in an aqueous solution (strictly speaking, this demonstrates a photoelectrochemical effect). A while later, Smith and Adams were the first to publish reports on photoconductivity, in 1873 and 1876, respectively, working with selenium. The use of this technology was not taken up until after further work, such as the development of the p-n junction in silicon electronics, leading to the discovery of the silicon-based solar cell.

The modern solar cells was been developed by D. M. Chapin and C. S. Fuller in 1954 at Bell Labs using a solid-state semiconductor junction (Chapin et al., 1954). Since the good quality silicon wafers can be produced in the 1950s, the silicon electronics became the main source materials for the PV industry. Its potential application in space exploration led to the first use of a solar cell being on the satellite Vanguard 1 in 1958.

**FIGURE 2:**
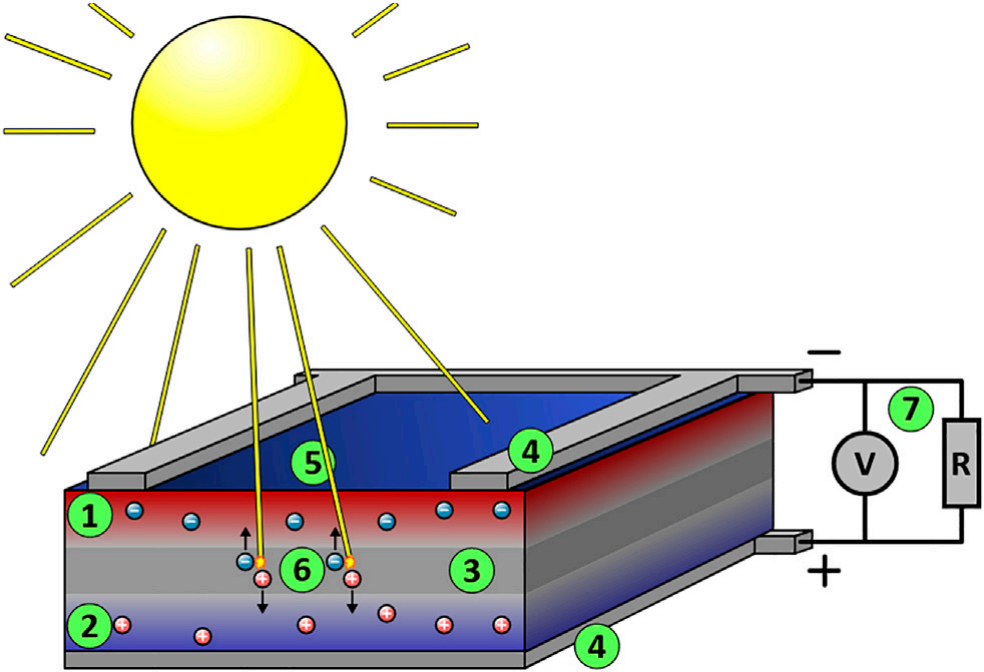
Schematic representation of a silicon monocrystalline solar cell. The circles present the excited electrons and holes. They are then extracted to the conductance band of an n-type conductor and from there passed around a circuit and eventually returned to the valence band of the p-type conductor. (1) The upper silicon layer is interspersed with electron donors (e.g., phosphorus atoms), negatively doped. There are too many electrons here (n-layer). (2) The lower silicon layer is interspersed with electron acceptors (e.g., boron atoms), positively doped. Here, there are too few electrons, i.e., too many defects or holes (p-layer). (3) In the border region of the two layers, the excess electrons of the electron donors bind loosely to the vacancies of the electron acceptors (they occupy the vacancies in the valence band) and form a neutral zone (p-n junction). (4) Since there is now a lack of electrons at the top and a lack of vacancies at the bottom, a constantly present electric field forms between the upper and lower contact surfaces. (5) Photons (light quanta, “sun rays”) enter the transition layer. (6) Photons with sufficient energy transfer their energy to the loosely bound electrons in the valence band of the electron acceptors in the neutral zone. This releases these electrons from their bond and lifts them into the conduction band as free charge carriers. Many of these free charge carriers (electron-hole pairs) disappear again after a short time through recombination. Some charge carriers drift—moved by the electric field—to the contacts in the similarly doped zones (see above); i.e., the electrons are separated from the holes, the electrons drift upwards, and the holes downwards. A voltage and a usable current are created as long as further photons continuously generate free charge carriers. (7) The “electron” current flows through the “outer circuit” to the lower contact surface of the cell and recombines there with the holes left behind (Paetzold, 2018).

Dr. Ching W. Tang reported the first organic cell and published his research results in 1986 (Tang, 1986). In 1991, Grätzel and O’ Regan found the first dye-sensitised photovoltaic device which has efficiency in the full sunlight of 7.1% (O’regan and Grätzel, 1991). Since then, Perovskite materials have been first incorporated into a solar cell as reported by Miyasaka et al. in 2009; this cell was based on a dye-sensitised solar cell and generated only 3.8% power conversion efficiency (Kojima et al., 2009). A power conversion efficiency of 16% at AM 1.5G one sun illumination was reported (Lee et al., 2014), and the efficiency increased rapidly beyond 20% with the developments of new functional materials and blends (Thomas and Thankappan, 2018). Solar cells technologies rely on the occurrence of the photovoltaic effect, which is the generation of a direct current electrical power from semiconductors when they are illuminated by photons. All solar cells require a light-absorbing material in the cell structure to absorb photons and generate free electrons by the photovoltaic effect. In a standard p-n junction solar cell, the light hitting the junction between a hole conducting and electron-conducting semiconductor can create electron-hole pairs which in turn leads to a potential difference across the interface (see Figure 2) (Luque and Hegedus, 2011).

It remains the case that the first generation solar cells remain largely still in production industrially during the first decades of the 21st century. These are based on single-junction silicon wafers, including common amorphous silicon systems, such as silicon carbide, silicon germanium, and silicon nitride. Crystalline silicon allows for an increase in the efficiency of the cell but at a cost (Parida et al., 2011); however, cost, in terms of £/W, remains the greatest barrier to further expansion of PV-generated power and cost reduction is the prime goal of the PV sector (Bagnall and Boreland, 2008).

Second generation solar cell technologies are based on thin-film technology that reduces the cost of the cell by reducing the amount of material required. These thin films are chemically or physically deposited on low-cost substrates such as glass creating a cheaper cell with similar efficiencies to the first generation cells (Soteris, 2018). The third generation is a step beyond the single-junction cells that include not only multijunction cells but also polymer and organic and dye-sensitised solar cells (DSSCs) (Dragonetti and Colombo, 2021).

Fundamentally, the basis of PV device construction relies on sensing technologies reliant on photodiodes. A diode is an electrical component that allows current to flow only in one direction. A photodiode is a type of photodetector which converts light into current or voltage, whereby an organic photodiode is made of highly conjugated organic material. If the electrodes of a photodiode are connected by a wire, in the dark, no current will flow however in light current flows from cathode to anode. When the material absorbs a photon, an exciton is formed. This is known as the inner photovoltaic effect. The exciton is then separated, holes move to the anode, and the electrons move to the cathode. Once they are separated, a photocurrent is produced.

**FIGURE 3:**
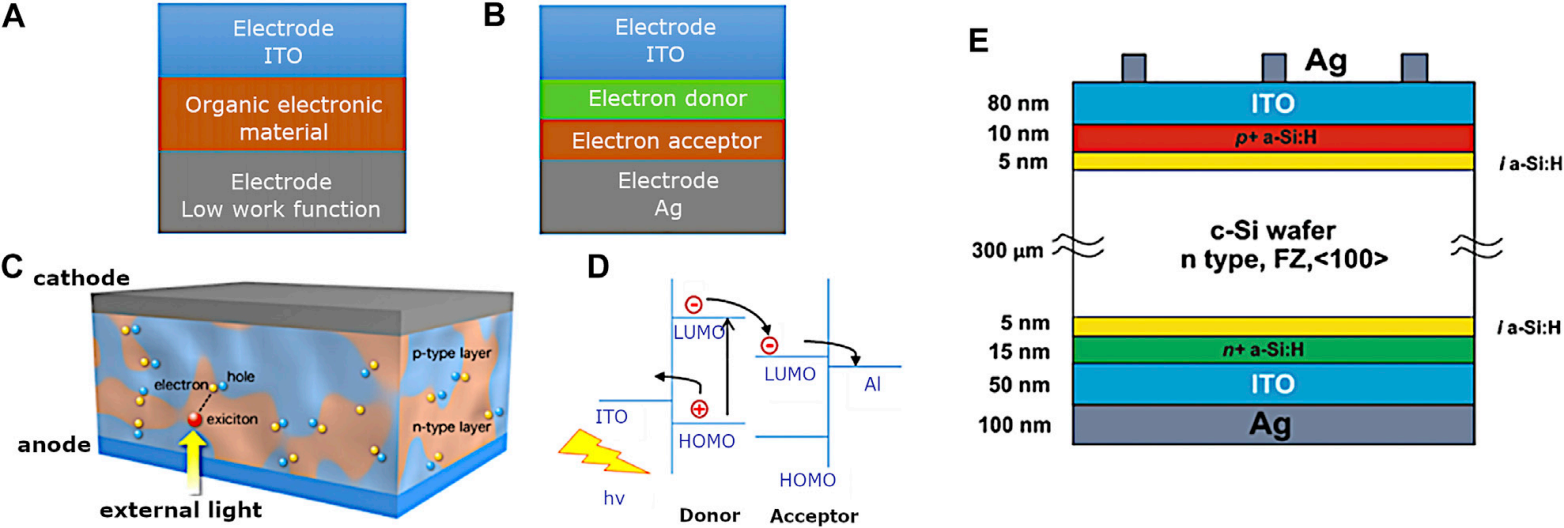
Schematic representation of **(A)** OPV photovoltaics, **(B)** heterojunction or bilayer photovoltaics, **(C)** bulk heterojunction photovoltaics (Organic Solar Cell (OSC) | Chihaya Adachi lab), **(D)** electron-hole movement inside the energy levels of the photovoltaics materials, and **(E)** schematic diagram of a silicon heterojunction solar cell.

Commonly encountered device structures include single layer OPV whereby the organic electronic material is sandwiched between a high work function electrode and a low work function metal, whereby the difference in work function establishes an electric field within the organic layer. The potential created by the two electrodes separates the exciton (Figure 3A).

Additionally, heterojunction or bilayer photovoltaics incorporate the two materials that have different electron affinities and ionisation energies. The differences need to be large so the electric field is strong enough to separate the exciton (Figure 3B). Bulk heterojunction PV incorporate devices whereby the acceptor and donor materials are mixed together to form a polymer blend. The working principle relies on light excitation generating an exciton, which is then broken at the interface between donor and acceptor material and the charges transfer to the electrodes. The open-circuit voltage is in turn dependent on the band gap between the highest occupied molecular orbit (HOMO) and the lowest unoccupied molecular orbit (LUMO), whilst the photocurrent is determined by the voltage. Excitons must diffuse to a p-n interface to separate. Small exciton diffusion length may be overcome by mixing donor and acceptor to shorten the distance to the interface. Morphology and phase separation of the blend are crucial to solar cell performance.

For another classification often used in the solar cells industry, the traditional silicon solar cells technique belongs to the first generation technologies. The first generation silicon solar cells use mono- or multicrystalline silicon as the main materials for the production of solar cells. Figure 3E shows a typical monocrystalline silicon solar cell. This kind of silicon solar cells is a single-junction solar cell in different regions; n-type or p-type semiconducting materials are doped (Bagnall and Boreland, 2008). The efficiency of these single-junction solar cells can rise up 20%. The improvements of silicon single-junction solar cells were addressed by silicon heterojunction solar cells (De Wolf et al., 2012; Qu et al., 2021). Figure 3E shows a typical structure of silicon heterojunction solar cell. Although the efficiency of this kind of solar cell is relatively very high in comparison with that of organic solar cells, the high cost and the pollution causing in the production process set back the development of the PV industry (Nelson, 2003).

The second generation solar cells incorporate the thin-film solar cells produce thinner layer crystals than the first generation materials film, made by depositing one or more thin layers or thin film of photovoltaic material on a substrate, such as glass, plastic, or metal. It was reported that using CdTe and CIGS (Copper Indium Gallium Selenide) thin film to generate single-junction solar cells, the efficiency of these thin-film solar cells can raise up to 21% (Pearce et al., 2007). However, they involve a technically demanding of high requirement for producing process and it was found that it is very difficult to transfer this technology from laboratory scale to commercial-scale products.

The third generation solar cells incorporate organic solar cells and several different types of DSSCs, and so on. The organic solar cells include Schottky-type solar cell, bilayer heterojunction solar cell, and bulk heterojunction solar cell (BHJ) (Hoppe and Sariciftci, 2004). The Schottky-type solar cells have a typical metal-organic-metal sandwich structure. From R. O. Loutfy and J. H. Sharp’s research, macrocyclic molecules such as porphyrins and phthalocyanines were considerable material choices for the organic layer present in the sandwich structure (Loutfy and Sharp, 1979). In 1985, Tang et al. first reported the bilayer heterojunction solar cells. The efficiency of the organic solar cell was then improved significantly. In his work, small molecules of copper phthalocyanine and a perylene tetracarboxylic derivative were used as the active layer of the solar cell (Tang, 1986). However, the diffusion of excitons only occurs in several nanometres near the interface of two organic materials in the bilayer heterojunction solar cell, which constituted one of the main limitations for the bilayer heterojunction solar cell. In order to solve this problem, bulk heterojunction solar cell, which relies on the mixing of the electron donor and acceptor materials together, was introduced (Hiramoto et al., 1992; Yu et al., 1995). The bulk heterojunction is an interpenetrating network of acceptor and donor where the phase separation is commonly between 10 and 20 nm, which is within the effective diffusion length of an exciton. The emergence of bulk heterojunction is a breakthrough in organic solar cell.

Alternative third generation solar cell is the dye-sensitised photovoltaic device, which was firstly founded in 1991 by O’regan and Grätzel (1991). Dye-sensitised solar cell combines organic and inorganic components together and assembly nanomaterial together to build up a multilayer structure. The dye molecules are working as a light-harvesting material in the cell structure to absorb photos and generate free electrons by the photovoltaic effect. Modern DSSCs are composed of a porous layer of titanium dioxide nanoparticles, covered with a molecular dye that absorbs sunlight. The titanium dioxide is immersed under an electrolyte solution, above which is a platinum-based catalyst. As in a conventional alkaline battery, titanium dioxide regarded as an anode and platinum regarded as a cathode are placed on either side of a liquid/solid electrolyte conductor.

**FIGURE 4:**
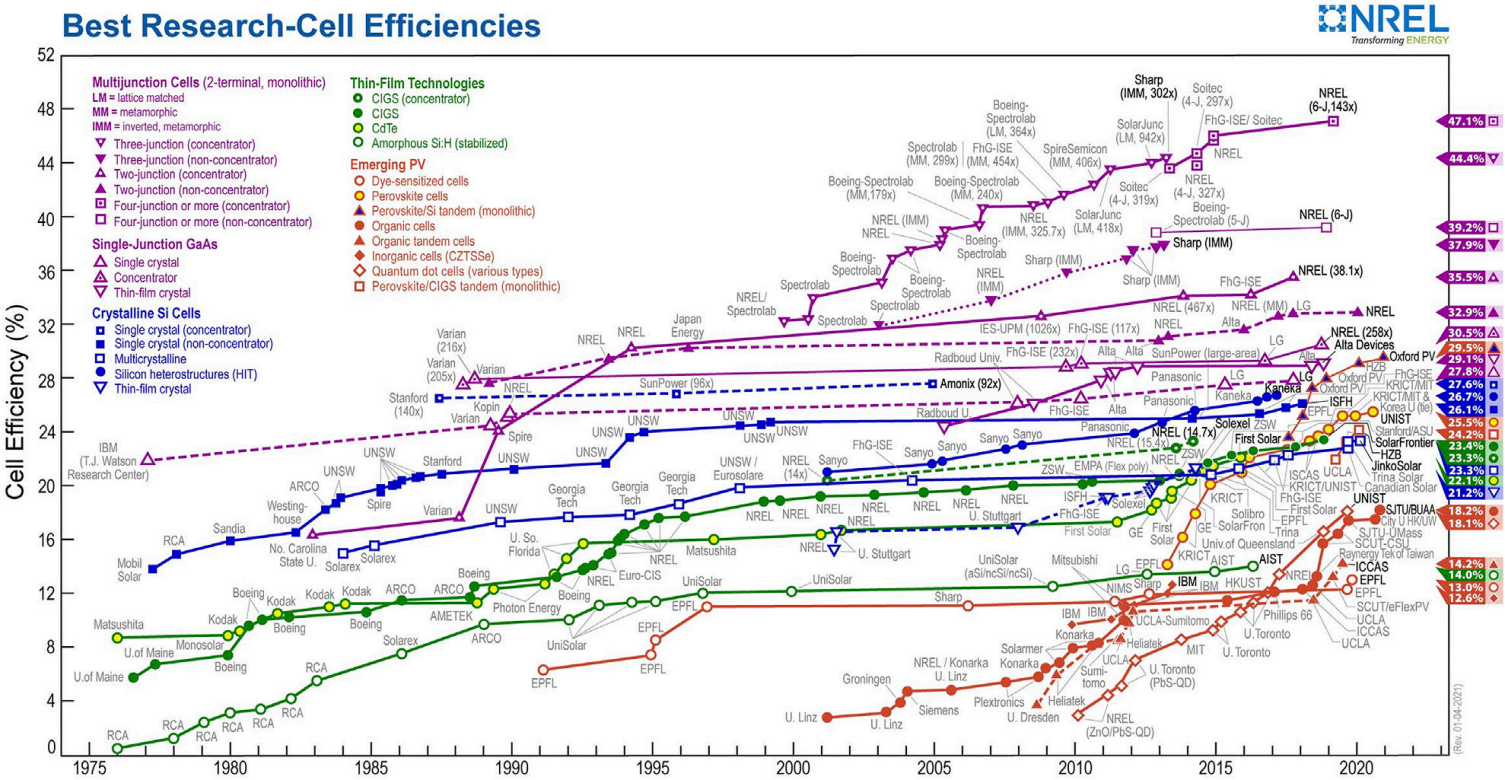
The efficiency of record-performance solar cells in each of the primary technologies. Amorphous Si (a-Si) and the single-crystal concentrator cells are triple junctions in the most recent devices whilst the others are single junctions. CIGS indicates the alloy Cu(In1−xGax)Se2 (Photovoltaic Research | NREL).

Devices assembly involve layering methods include spin coating, blade coating, drop casting, and evaporation either by thermal or electron beam. Developments showed a departure from the classical solid-state junction device, by replacing the phase in contact with the semiconductor by an electrolyte (liquid, gel, or organic solid), thereby forming a photoelectrochemical device. Figure 4 shows a comparison of the efficiencies of the different types of solar cells; this presents the efficiency of recorded best research solar cells performance, generated by the National Centre for Photovoltaics in the United States.

Perovskite solar cells are a relatively recent discovery, where a solid-state material such as a perovskite was first used as a light absorber (Kojima et al., 2009). These are adaptations of dye-sensitized solar cells, the fact of continuing to use organic dyes, in technical terms, limits the use of this range of solar cells based on cheap and readily available materials. The most commonly studied perovskite absorber is methylammonium lead trihalide (CH_3_NH_3_PbX_3_, where X is a halogen ion such as I^−^, Br^−^, and Cl^−^), with a bandgap between 2.30 and 1.57 eV depending on halide content. It was first used as a replacement of the dye in DSSCs in order to solve the problem of limited light harvesting of organic dye (Kojima et al., 2009). However, the perovskite was found to easily dissolve or decompose in the liquid electrolyte, and it even degraded in a few minutes when it was first designed. A solid-state hole-transporting conductor was then applied to solve the instability of perovskite. In 2021, researchers at South Korea’s Ulsan National Institute of Science and Technology (UNIST) and the Swiss Federal Institute of Technology Lausanne (EPFL) have achieved a new record conversion efficiency of 25.6% in a single-junction perovskite solar cell (Jeong et al., 2021).

### A Deeper Incursion Into DSSCs

**FIGURE 5:**
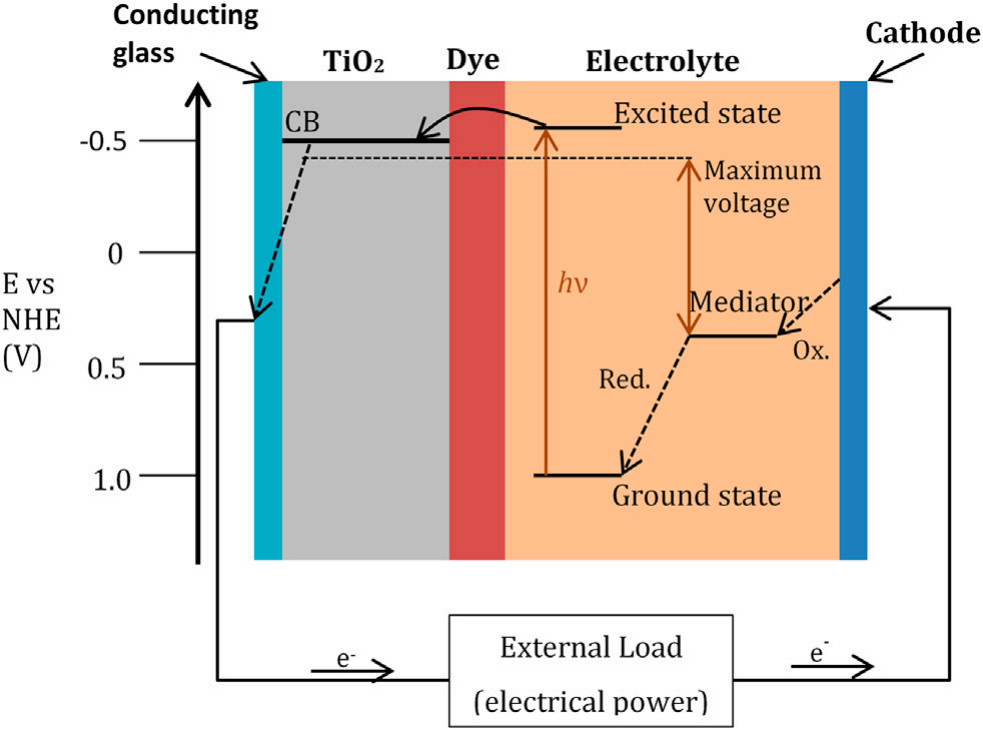
Schematic representation of the operation principle for a typical dye-sensitised nanocrystalline solar cell. Photoexcitation of the sensitiser from the ground state to the excited state is followed by electron injection into the conduction band (CB) of a semiconductor oxide film. The dye molecule is regenerated by the redox system, which itself is regenerated at the counter electrode by electrons passing through the load. Potentials are referred to as the normal hydrogen electrode (NHE). Image adapted with permission from Grätzel (2005). Copyright 2005 American Chemical Society.

The prototype for this family of devices was discovered by Grätzel at the Ecole Polytechnique Fédérale de Lausanne which involved using ruthenium-based dyes adsorbed onto nanocrystalline films of titanium dioxide. The ruthenium dye serves as a p-type conductor and absorbs the photon producing an excited electron which is then injected into the conductance band of the n-type conductor (TiO_2_). To complete the circuit, the dye must be regenerated by electron transfer from a redox species at the counter electrode, usually an iodide/triiodide electrochemical couple (Figure 5). The initial experiments were very successful, with up to 80% of the incident photons being converted into electrical current and an overall light-to-electricity yield of 7.1–7.9% (O’regan and Grätzel, 1991). The electrons injected into the solid permeate very rapidly across the TiO_2_ layer and during this diffusion of electrons maintain their high electrochemical potential equal to the quasi-Fermi level of the semiconductor under illumination. Thus, the principal function of the oxide, apart from supporting the sensitiser, is that of charge collection and conduction. Although TiO_2_ has been the material of choice, alternative wide-bandgap oxides such as ZnO and Nb_2_O_5_ have also been investigated (Grätzel, 2005). The dye used in these cells has to withstand the conditions encountered in the practical application of the solar cell and remain serviceable for 20 years, corresponding to 50–100 million turnovers for the dye (Grätzel, 2005).

Enhancing the efficiency of a DSSC is critically dependent upon the charge separation processes, whilst at the same time reducing the energetic losses required to drive these reactions. Addressing this challenge is fundamental to the challenges of enhancing the voltage output of devices and of utilising sensitiser dyes with lower optical bandgaps and therefore enhanced spectral overlap with the solar spectrum.

A further challenge is to move towards materials which can achieve similar device performance but with enhanced stability and/or processability, they must also have high light-to-electricity conversion efficiencies, ease of fabrication, and low production costs (Bessho et al., 2010). There is an increasing appreciation that meeting these challenges is a multidimensional problem, where any one materials change impacts upon several processes within the device (Listorti et al., 2011).

### Organic Bulk Heterojunction Solar Cells

Organic materials have the advantage of being cheap and easy to process, as well as being flexible compared to most inorganic sensitisers. The choice of materials is also practically unlimited, and specific parts of the solar spectrum can be selectively absorbed. Mimicking the natural light harvesting in photosynthesis, in which a number of chlorophylls and carotenoids are involved in light collection, suggests that it is likely that optimal photosensitisation in DSSCs will only occur using a mixture of dyes (Campbell et al., 2004; Radivojevic et al., 2012).

The removal of a metal-based system to a more flexible organic-based system leads to several advantages including the following:

• Low weight and flexibility of the PV modules.
• Semitransparency.
• Easy integration into other products.
• New market opportunities, e.g., wearable PV.
• Significantly lower manufacturing costs compared to conventional inorganic technologies.
• Manufacturing in a continuous process using printing tools.
• Short energy recovery times and low environmental impact during manufacturing and operations (Scharber and Sariciftci, 2013).

This type of solar cells has the potential not only to overcome the inherent cost problem of current cells but also to move the technology into new markets. Despite these advantages, organic cells are still considerably less efficient than single-crystal gallium, arsenide, or silicon, although one way these efficiencies can increase is through the increase in the number of p-n junctions, like in most third generation cells (Green, 2006). The difference here however is that bulk donor-acceptor heterojunctions can be formed simply by blending two organic materials, one serving as an electron donor (p-type conductor) and the other an electron acceptor (n-type conductor). This means that the distance between the two materials is reduced into the nanometre range, overcoming the unfavourable ratio of exciton (a bound electron-hole pair) diffusion length to optical absorption length (Sauvé and Fernando, 2015).

**FIGURE 6:**
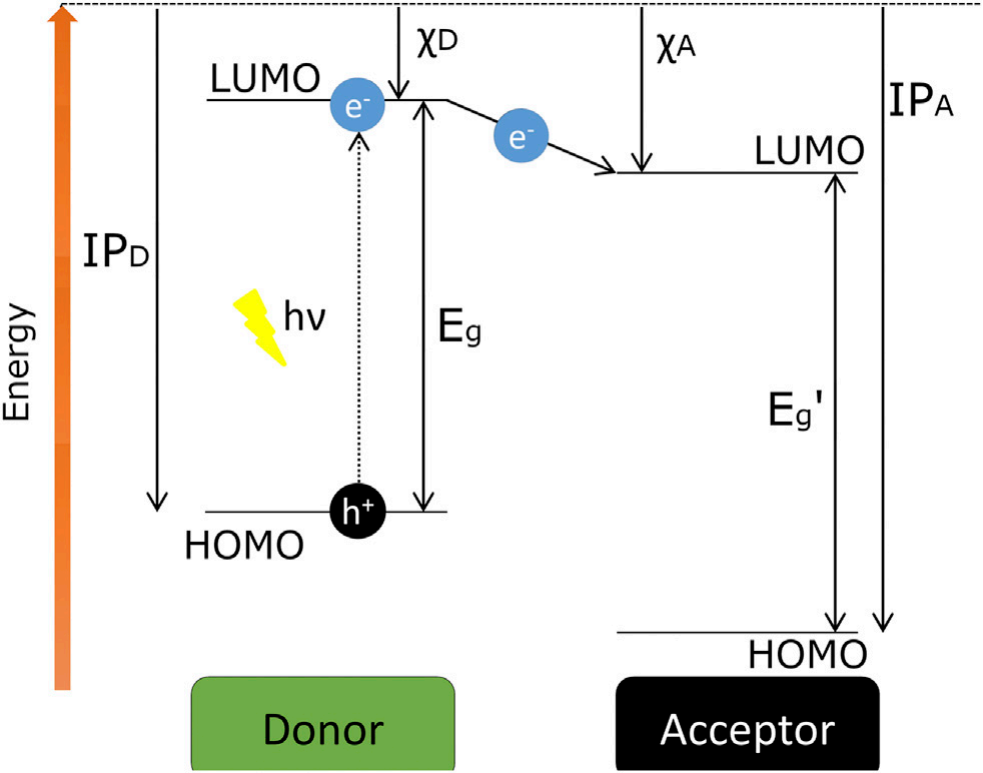
Representation of the energy levels diagram of a donor-acceptor system, where IP is the ionisation potentials, χ is the electron affinity, and E_g_ is the bandgap energy. The arrow between the LUMO levels indicates the photoinduced ET, which is the first step for generating free charge carriers. Adapted from Reference Scharber and Sariciftci (2013).

To be effective, when an exciton is produced by absorption of light, the pair must reach the junction and there dissociate into two free charge carriers (Figure 6). However, excitons typically diffuse only a few nanometres before recombining. Light is absorbed (and generates excitons) throughout the composite material, so the smaller the distance between each junction is, the more chance the exciton has of reaching the junction before recombination. Hence, photoinduced charge separation can occur very efficiently (Grätzel, 2001), and crucial to this is the understanding of the donor-acceptor interactions that are taking place between these junctions.

Due to the low dielectric constant of most organic materials, there is a strong Coulomb attraction between the electron-hole pair and so dissociation of the exciton at ambient conditions is very unlikely. A simple model on an electron-hole pair separated by 1 nm with a dielectric number of 3–4 would have a binding energy of 0.35–0.50 eV. This binding energy exceeds the thermal energy at room temperature by an order of magnitude and electron acceptor molecules need to be added to an organic semiconductor donor to facilitate the generation of free charge carriers (Scharber and Sariciftci, 2013). It is for this reason that an acceptor molecule is required to be the driving force behind the production of free charge carriers with the energy difference between the LOMOs of the two molecules being the predominant factor (Sariciftci et al., 1992).

The construction of supramolecular structures of π-conjugated molecules via self-assembly has been recognised as an important approach to manipulate their optical and electronic properties to generate “supramolecular electronics.” Due to the relatively short (<1 ns) exciton lifetime in organic semiconductors, quantitative charge generation requires very fast charge separation. To achieve efficient charge generation, excitons must be generated within their diffusion length to the nearest donor-acceptor interface relative to their lifetime. Recent measurements indicate that this diffusion length is in the range of 10 nm for several prototype conjugated polymers used in bulk heterojunction solar cells, which means that an intermixing of the donor and the acceptor moieties on the nanometre scale is required (Mikhnenko et al., 2012).

**FIGURE 7:**
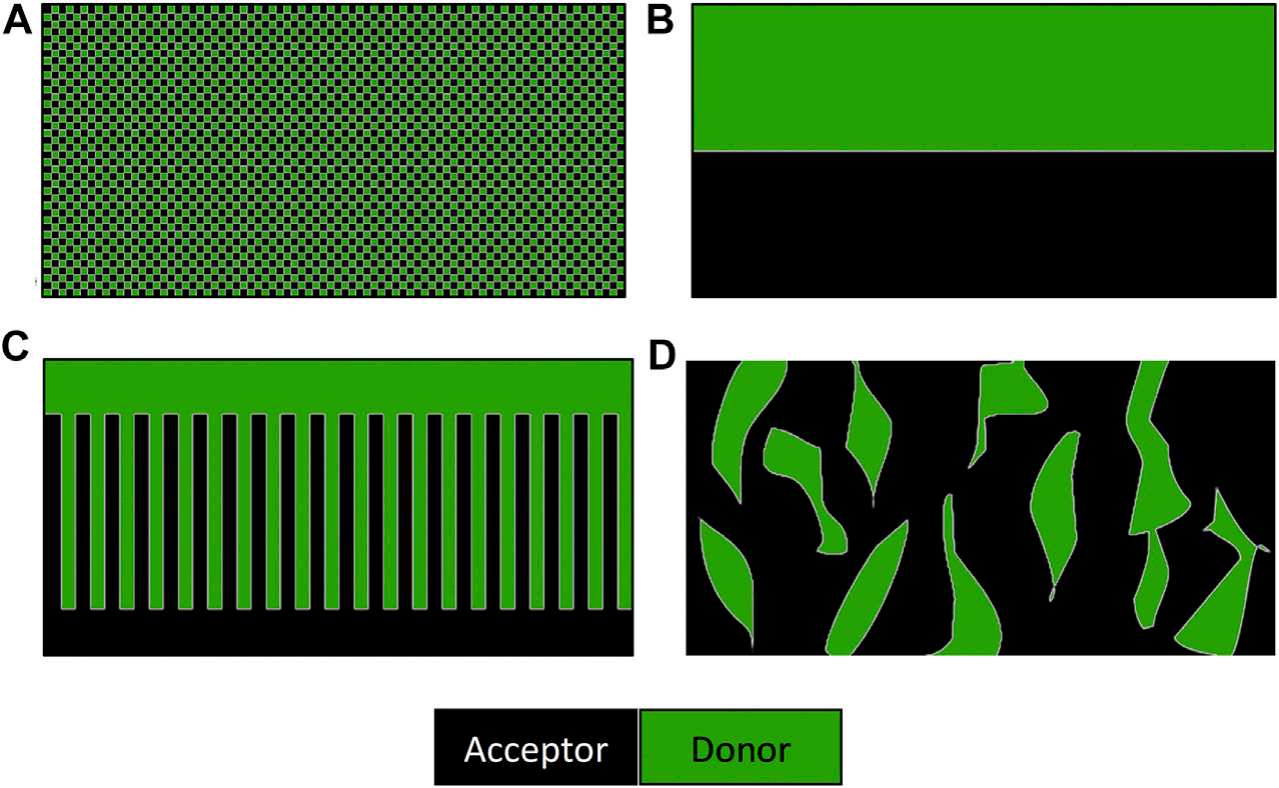
Schematic representation of the cross-section of the nanomorphologies of bulk heterojunction solar cells. **(A)** Fine mixture of donor and acceptor molecules, **(B)** bilayer arrangement, **(C)** ideal morphology of a bulk heterojunction solar cells, and **(D)** typical morphology of a solution processes device (Scharber and Sariciftci, 2013).

There is still no full consensus on how the ideal nanomorphology of a bulk heterojunction cell should be arranged. A very fine dispersion of the acceptor in the donor material, shown in (a) in Figure 7, would lead to efficient charge generation but poor charge transport. Ideal charge transport could be achieved by arranging the donor and acceptor in by bilayer stack (b). However, charge generation overall will be poor as it only happens at the interface between the two. Calculations and morphology simulation work have suggested that the arrangement shown in (c) should lead to ideal performance (Watkins et al., 2005). In addition to this loss of efficiency due to the recombination of the excitons or charge-transfer states prior to separation (called geminate recombination), further losses in efficiency have been shown to be caused by the recombination between pairs of dissociated electrons and holes, where each is generated by a different absorption event (called nongeminate recombination) (Dibb et al., 2011).

Bulk heterojunction (BHJ) solar cells offer a promising, low-cost, large-area, flexible, light weight, clean, and quiet alternative energy source for both indoor and outdoor applications. Table 1 shows the comparison of the efficiencies of different types of solar cells, showing that BHJs are not far off the efficiency of more common inorganic based cells. What is more, due to the wide range of organic dyes that are available for these cells, the application can be thought of as more than just a functional energy device, especially when considering the colour and relatively thin width of the final cell.

**TABLE 1:** Comparison of recently reported cell efficiencies for traditional solar cell types.

Classification	Efficiency (%)	References
Si (crystalline)	25.6 ± 0.5	Green et al. (2014)
Si (multicrystalline)	20.4 ± 0.5	Schultz et al. (2004)
Si (microcrystalline)	11.0 ± 0.3	Petermann et al. (2012)
Si (amorphous)	10.1 ± 0.3	Green et al. (2014)
GaAs (thin film)	28.3 ± 0.9	Green et al. (2014)
Dye sensitised	11.9 ± 0.4	Komiya et al. (2011)
Bulk heterojunction	10.7 ± 0.3	Scharber and Sariciftci (2013)

This tuneable colour leads to photonic nanostructures incorporated with photovoltaics capable of producing desirable colours in the visible band and utilise the absorbed light to simultaneously generate electrical powers. In contrast to the traditional colourant-based filters, these devices offer great advantages for electrooptic applications (Park et al., 2011).

Improvements in efficiency and stability, namely, minimising loss mechanisms and improving light harvesting, are required to commercialise this technology (Werner et al., 2010). These organic dyes are also less stable in common working conditions, in high-temperature and high light environments. Although it has been found that some changes to the donor molecule can increase the stability (García-Iglesias et al., 2011), there is still more to be done to create an efficient and stable organic molecule for light-harvesting applications.

Organic solar cells, as one of the most studied types of PVs to date, are based on donor-acceptor heterojunctions and attract increasing interest due to the advantages of light weight, low cost, and flexible as well as due to the fact that a vast range of materials with tuneable band gaps are available (Brabec et al., 2001; Chen et al., 2013). Organic solar cells were first discovered due to the study of the perylene-iodine complex in 1954 (Akamatu et al., 1954). As described in the previous section, the organic solar cell is limited by the low dielectric constant of organic semiconductor, which leads to the slow mobility of electrons and holes and the exciton diffusion length of the charge carrier is significantly limited. It is reported that the diffusion length of organic semiconductor is believed to be 5–20 nm (Lunt et al., 2009). As a result, the thickness of phase separation and the active layer should be extremely carefully controlled. When this is reduced into nanometre ranges, the interface between donor and accepter materials is enhanced. Thus, new forms of donor-accepter blended structures can improve the efficiency of PV.

**FIGURE 8:**
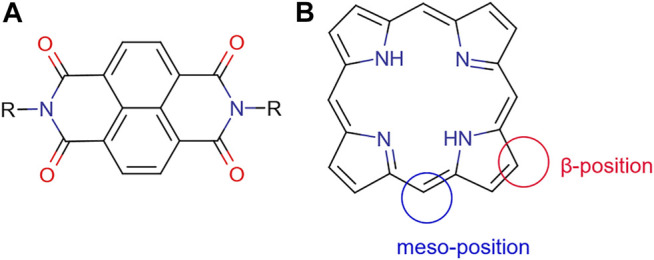
**(A)** General molecular structure of naphthyl diimides (NDIs); **(B)** framework structure of a free-base porphyrin presenting *meso*-position and *β*-position, which may be functionalised. Additionally, the N*H*’s are available for deprotonation and subsequent metallations.

In bulk heterojunction solar cells, the donors are typically organic systems having an electron-rich structure, whilst the acceptor normally shows conjugated π bonds, which due to the electron affinity can be the active part with the role to transport electrons (Figure 8). Another important factor for donor-acceptor is the energy level of the donor and acceptor, which should be well matched. As a result, ideally, in organic bulk heterojunction solar cells, the LUMO of the donor systems should be at least 0.3–0.4 eV higher than the acceptor’s LUMO energy level, which is needed to address the efficient exciton dissociation (Mishra and Bäuerle, 2012). At the same time, the bandgap between the donor’s HOMO energy level and the acceptor’s LUMO energy level should not be too large to become positive to electron exchange. The bandgaps between donor HOMO energy level and the acceptor LUMO energy determine the open-circuit voltage and a big energy gap can lead to an energy loss, causing a lower open-circuit voltage for the resulting solar cell.

To optimise the performance of a selection of PVs, donor and acceptor material combinations have been studied and tested so far (Sariciftci et al., 1992). Compared with the use of polymers, small molecules were studied for bulk heterojunction solar cells assembly over a longer period of time, due to the following advantages: relatively easy to prepare and purify and offer a significantly improved reproducibility.

More importantly, with the advancement of supramolecular chemistry, the modification of small molecule of the classes listed in Table 1.1 becomes accomplishable (Mikhnenko et al., 2012). For instance, changing the variety or adding functional side groups became possible, which means that the HOMO and LUMO energy level of the small molecule can be easily tuned. This modification is very important for bulk heterojunction solar cells production and design of other solar cells incorporates small molecule in their systems. It means that solar cells can be designed and optimised not only at the assembly stage but also from the start, despite the careful choice of the material. The small molecule can be classified by their working role in bulk heterojunction solar cells: donor, acceptor, and acceptor/donor dyad, which can simultaneously function as either donor or acceptor depending on the environment (Zhang et al., 2018). Table 2 shows the common small molecule used in organic solar cells.

**TABLE 2:** Small molecules used in organic solar cells.

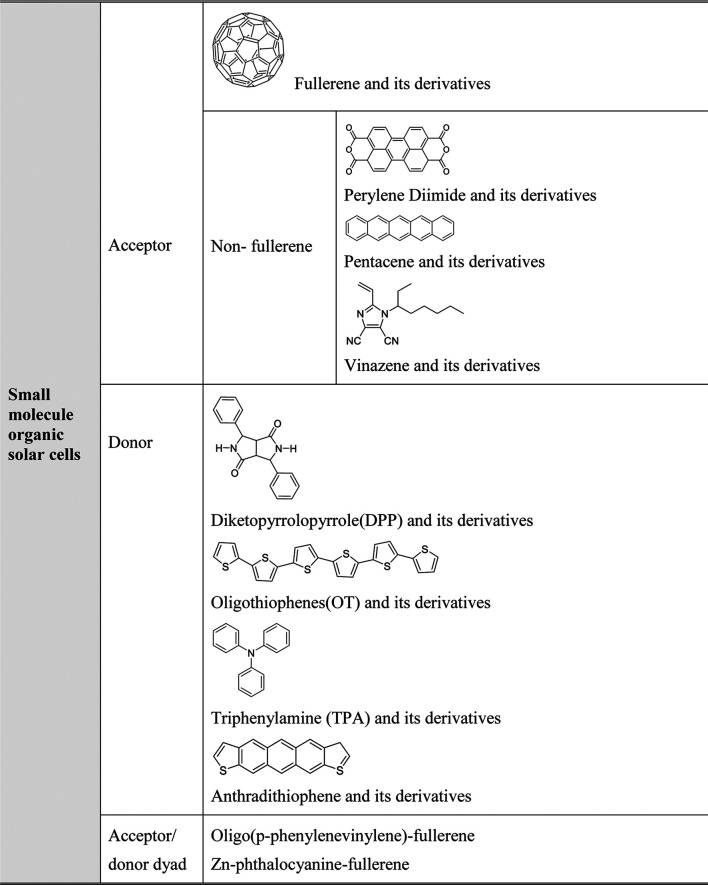

As shown in Table 2, acceptor materials used in organic solar cells can be classified into two groups: one is fullerene and its derivatives, and the other one includes nonfullerene and functional derivatives. From the class of fullerene and its derivatives, PC_71_BM (molecule structure shown in Table 3) shows an impressive performance for its good electron mobility and solubility (Ganesamoorthy et al., 2017).

**TABLE 3:** Small molecule based on fullerene and its derivatives used in organic solar cells and their structure.

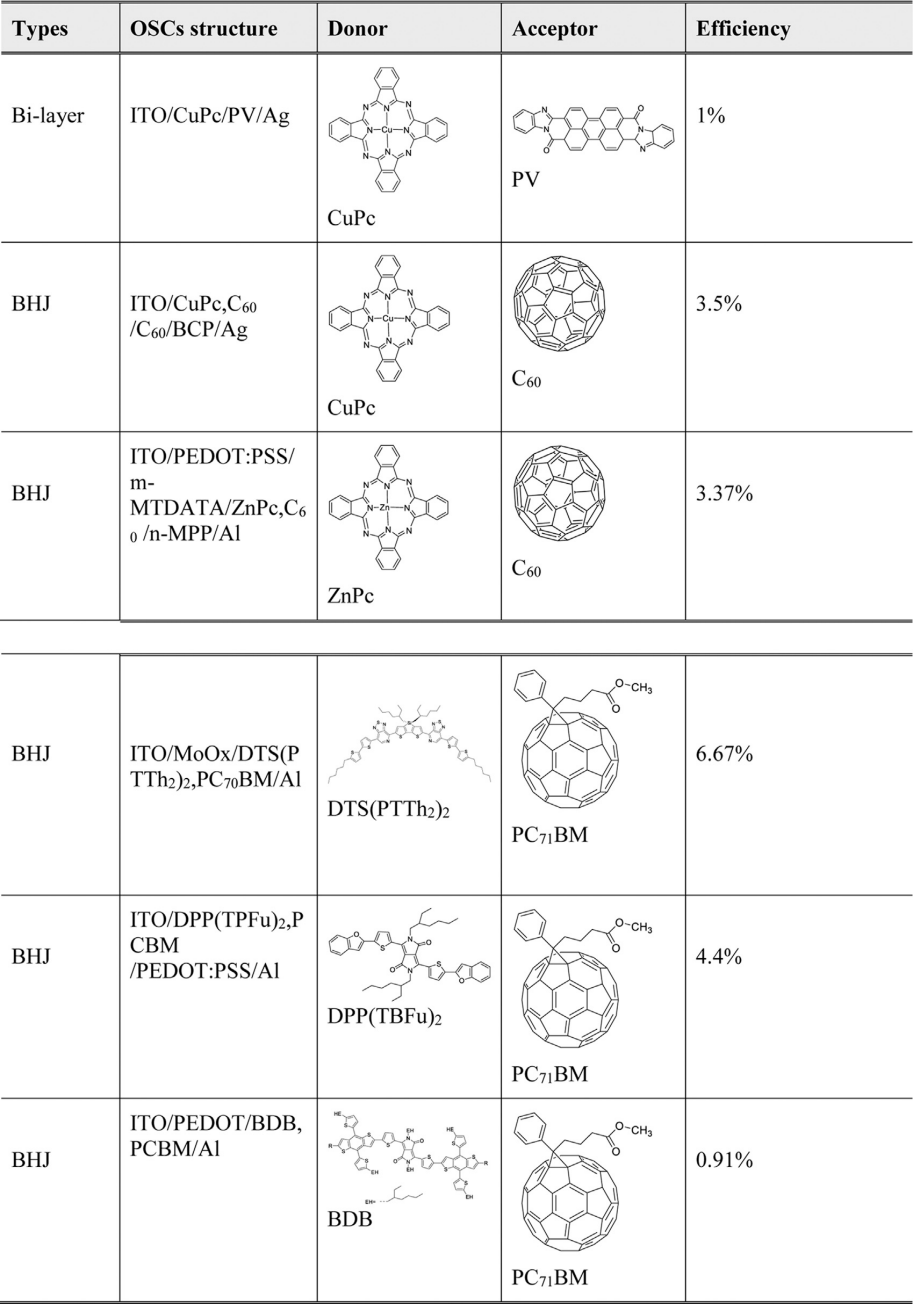

Recently, the research of acceptor dyad and triad systems based on perylene tetracarboxylic diimide and fullerenes also show promising properties (Chamberlain et al., 2011b). For donor materials, phthalocyanine (Pc) and its metallations present good property in light absorption and had been applied in the first bilayer structure. Also reported by Uchida, a homogeneous layer of CuPc and C_60_ homogeneous layer, the active layer corresponds with a C_60_ and 2,9-dimethyl-4,7-diphenyl-1,10-phenanthroline cathode. This system exhibits a 3.5% efficiency (Uchida et al., 2004). Diketopyrrolopyrrole (DPP) and its derivatives donor materials also display good properties. A 4.4% efficiency and 0.9 eV open-circuit voltage were reported as using DPP (TBFu)_2_ as donor and PCBM as acceptor (Walker et al., 2009). Recently, Zhang reported using modified DPP in organic bulk heterojunction solar cells to enhance the hole mobility from 4.14 × 10^–4^ to 7.75 × 10^–3^ cm^2^ V^−1^ s^−1^ and raise the fill factor from 27 to 57% when blended with PC71BM (Zhang et al., 2014). Table 3 exhibits some of the small molecules in organic bulk heterojunction solar cells.

### Dye-Sensitised Solar Cells (DSSCs)

Dye-sensitised solar cells-based technologies (DSSCs) are widely studied and longstanding candidates for the current and next generation of solar cells (NREL; Rockett, 2010; Sharma et al., 2018). As mentioned devices above, although it was Edmond Becquerel who discovered the photovoltaic effect (which describes the conversion of sun light into electricity), it was not until 1991 when Grätzel and O’Regan reported the first modern dye-sensitised photovoltaic device, thus introducing a mesoporous semiconductor electrode with a high internal surface area which should be an efficiency in the full sunlight of 7.1% (O’regan and Grätzel, 1991). This discovery led to a paradigm shift in the fields of photoelectrochemistry and photovoltaics in general (Grätzel, 2005). Before the report on Grätzel and O’Regan work, previous efforts to develop DSSCs all failed due to the fact that there was no smooth semiconductor surface introduced, specifically that of the TiO_2_ thin film, in the system. In 2006, Y. Chiba et al. reported a DSSC with an efficiency of 11.1% (Chiba et al., 2006). A typical DSSC includes four major components: photoelectrode, dye molecule, electrolyte, and counter electrode.

Compared with other types of solar cells, the DSSCs have several different advantages. First of all, the cost of manufacturing a DSSC is relatively low since these combine simple synthetically available organic and inorganic components, which are assembled on the nanoscale leading to the build-up of complicated hybrid structures. Additionally, the fabrication process of DSSCs is relatively simple compared with conventional p-n semiconductor solar cells, where electron-hole pairs are generated in the bulk material and then there is a need for these to diffuse to the p-n interface in order to be extracted. The charge generation in DSSCs only takes place at the material surface; as a result, the requirement for material purity is dramatically reduced. Furthermore, the fabrication process is relatively simple despite the need for specialised equipment; it does not require a high vacuum, ultrahigh temperatures, and processing in a cleanroom (Ito et al., 2008). Due to their layered structure, the DSSCs can be generated by using a printing technique, which makes them accessible to an industrial-scale generation of such device (Ito et al., 2007). The operating principles of DSSCs rely on the following processes: 1) dye molecule photoexcitation, 2) electron ejection, 3) regeneration of dye molecule with electrolyte, 4) recombination of TiO_2_, and 5) regeneration of electrolyte.

One of the most commonly used wide-gap semiconductors for DSSCs photoelectrodes is TiO_2_ due to the fact that this is a material which is stable and nontoxic and has an energy gap of ∼3.0 eV. An important requirement for the photoelectrode semiconductor is the high transport mobility of the charge carrier, which is needed to reduce the electron transport resistance (Petermann et al., 2012). As an alternative, ZnO having different nanostructures has been actively been studied in this context due to its similar bandgap and conduction band edge (Matsumura et al., 1977; Matsumura et al., 1981; Jose et al., 2009). Yang et al. also reported a dense array of oriented crystalline ZnO nanowires with a surface area up to one-fifth of that of TiO_2_, which had a sun conversion efficiency of 1.5% (Zhang and Cao, 2011). Compared to similar ZnO-based devices with power efficiency of up to 1.6%, ZnO nanotube-based cells showed exceptional photovoltage, but the higher surface area may absorb excess dye molecules (Martinson et al., 2007). Besides ZnO, some other binary metal oxides, such as Fe_2_O_3_, ZrO_2_, Nb_2_O_5_, Al_2_O_3_, and CeO_2,_ and ternary compounds, such as SrTiO_3_ and Zn_2_SnO_4_, have been also studied and tested as photoelectrodes in DSSC, but their efficiency is significantly lower with respect to that of TiO_2_ (Moharam et al., 2021).

Along with the photoelectrode, a key component of the DSSC is the photosensitiser, a dye molecule, which functions as a light absorber and injects electrons into the conduction band of the photoelectrodes. To be regarded as a competitive dye sensitiser, a molecule should have good solubility in a range of organic solvents, strong light absorption in the visible and near-IR region. The photosensitiser should have anchoring groups, such as -COOH, -H_2_PO_3_, and -SO_3_H; their role is to bind the dye strongly onto the semiconductor surface. The dye of choice must have good thermal stability and good chemical stability and more importantly, it should also have suitable HOMO and LUMO energy levels to match with other molecular components used in the same DSSCs (Carella et al., 2018). The dye molecule design is made that it does not favour its self-stacking/self-aggregation. It was also reported that, through optimisation of the molecular structure of the dye or by addition of coabsorbers that prevent aggregation, the unfavourable dye aggregation on the semiconductor surface could be avoided and the cell performance can be improved (Mann et al., 2008).

According to reports, coordination complexes of Ru and Os supported by squaraines, porphyrins, phthalocyanines, perylenes, pentacene, cyanines, and coumarins can be used as competitive dye molecules (Hagfeldt et al., 2010). It was reported that the most efficient (>10%) DSSCs incorporate the ruthenium polypyridyl complex N3 and a similar structure slat (Bu_4_N)_2_ [Ru (4-carboxy, 4-carboxylato-2,2-bipyridine)_2_(NCS)_2_] (N719) (Nazeeruddin et al., 2005; Chiba et al., 2006). However, ruthenium is toxic and in low abundance on Earth. Furthermore, ruthenium-based dye molecules are not sustainable options likely to afford the large scale solar cells development in the long term. As stated above, some other types of small organic dyes molecules have also been tested in DSSCs: 9% efficient with indoline (Ito et al., 2006); 6.5% efficient with coumarin (Wang et al., 2007); 5.2% efficient with hemicyanine (Chen et al., 2005); 4.5% efficient with squarine (Yum et al., 2007); 7.1% efficient with porphyrin (Campbell et al., 2007); 3.5% efficient with phthalocyanine (Cid et al., 2007). Rather than changing the type of the supporting ligand and molecule, the substitution of the coordinating metal centre has also been used in dye molecule optimisation (Armel et al., 2011).

As another electron transfer part, the electrolyte plays a very important role in the DSSCs by facilitating the transport of charge between the dye molecule and the counter electrodes. The ideal liquid phase electrolyte and its solution should have low viscosity, negligible vapour pressure, high boiling point, and high dielectric properties. Additionally, factors like robustness, environmental sustainability, and ease of processing also need to be considered prior to DSSCs industrial fabrication. As initially observed by Grätzel for his systems, all reports of efficient DSSCs to date (>4% at 1 sun illumination) have utilised the I_3_^−^/I^−^ couple as the redox shuttle of choice. The good performance of I_3_^−^/I^−^ in these cells was due to the attribution of efficient dye regeneration combined with exceedingly slow electron transfer from TiO_2_ to I_3_^−^. For example, when I_3_^−^/I^−^ was employed with compound N3, the regeneration yield was found to be quantitative. In addition, loss of electrons *via* interception by I_3_^−^ is at short-circuit and can be negligible, which allowed photoinjected electrons to be collected with near-unity efficiency (Clifford et al., 2007). Other attempts were also made to find an alternative redox system in DSSCs, for instance, Br_3_^−^/Br^−^ (Wang et al., 2005b), Co^2+^/Co^3+^ (Nusbaumer et al., 2001), Fe^2+^/Fe^3+^ (Tian and Tatsuma, 2005), triethanolamine (Nakanishi et al., 1998), two pseudohalogen couples (SeCN)_2_/SeCN^−^ and (SCN)_2_/SCN^−^ (Oskam et al., 2001), and other mixed systems of redox couples. Water-based electrolytes for DSSCs have also been investigated (Murakami et al., 2003b; Law et al., 2010).

Additionally, naphthyl diimides (Figure 8) have been explored in the context of organic substrates for PV assembly on basis of their tendency to form n-type over p-type semiconductor materials (Katz et al., 2000). They are versatile materials capable of self-assembly with other aromatic species such as graphene which make them ideal for donor-acceptor systems and are photoactive; also recent microwave technologies gave rise to functional NDIs available in high yield and high purity and easy to isolate. Research led to halogen-modified structures, to study the effect on binding substrates and predict possible device assembly capabilities and performance (Hu et al., 2012; Tyson et al., 2016b). Studies on the incorporation of NDIs in a BHJ OPV in conjunction with a standard p-type material are underway (Rundel et al., 2017; Valero et al., 2020).

As the generation of solar cells developed from both the perspectives of theory and fabrication techniques, the structural improvement of DSSCs benefited from some emerging new ideas of cell design. The evolution of the solar cell technology forms the original idea of the electrolyte-based mesoscopic DSSC (introduced by Grätzel and O’Regan), and after the attempts of replacing the electrolyte with an organic p-type hole conductor, solid-state DSSCs (ssDSSC) were emerged (Murakoshi et al., 1997; Bach et al., 1998). The extremely thin absorbers (ETA) cell as introduced as the dye molecule is replaced with a semiconductor layer (Lévy-Clément et al., 2005; Kamat, 2013). For the *meso*-superstructured solar cell (MSSC), the ETA layer was replaced by a perovskite absorber and the n-type TiO_2_ layer was replaced with a porous insulating scaffold (Lee et al., 2012).

As stated above, the perovskite solar cells and DSSCs are following the similar operating principles but they differ in terms of choice of materials and structure. From a recent review by Henry J. Snaith (2013), three future directions for the DSSCs and perovskite solar cells technology were proposed: firstly, the Al_2_O_3_ could be removed but the perovskite is directly structured to give a porous film which can be subsequently filled with a charge conductor, giving a porous perovskite distributed p−n heterojunction solar cells. Secondly, thin-film p−i−n perovskite solar cells could be addressed, where no porosity is required and the device takes on an intrinsic or ambipolar structure where a thin perovskite film is sandwiched between p- and n-type charge-extracting contacts (Babu et al., 2020). The third possibility includes semiconductor MSSCs, where any solution-processed semiconductor, such as SbS (Itzhaik et al., 2009), can be structured by the porous scaffold to deliver the *meso*-superstructured materials. These systems are all currently processed in parallel with current developments in DSSCs.

## Porphyrins as Solar Energy Absorbers

One of the most attractive strategies is the development of organic solar cells that mimic natural photosynthesis in the conversion and storage of solar energy (Hasobe et al., 2005). For this application, porphyrins, with their extensive adsorption throughout the visible spectrum, show great promise as light-harvesting sensitisers for solar cells. Their high electronic excitation energy, typically exceeding 2.0 eV, powers a strong electron transfer, allowing a good conversion between light and chemical/electrical energy (Werner et al., 2010). One of the most attractive strategies is the development of dye molecules in organic solar cells, given that these mimic natural photosynthesis processes in the conversion and storage of solar energy. As chosen by nature, chlorophylls in plants function as antennae specifically designed to harvest light for the conversion of solar energy in complicated photosynthetic processes. Inspired by natural photosynthesis, scientists already utilised artificial chlorophylls model components, the porphyrins, as efficient light-harvesting centres due to their capability to absorb light and convert to electric energy in solar cells (Eichhorn et al., 1995).

Porphyrins are a group of heterocyclic macrocycle organic compounds, composed of four modified pyrrole subunits interconnected at their α carbon atoms via methine bridges (=CH−) (Figure 8). The porphyrin macrocycle rings have rich redox chemistry and a highly delocalised π-electron system; this structure makes porphyrin useful in both photosynthesis and respiration (Kadish et al., 1999). Porphyrins have also attracted a great deal of attention because of their strong Soret (400–450 nm) and moderate Q-bands (550–600 nm) absorption properties as well as their photochemical and electrochemical stabilities, known synthetic process, and handy control of redox potential by metallation (Xiang et al., 2011). For photochemistry applications, the porphyrin framework provides high electronic excitation energy, normally exceeding 2.0 eV, and this can power a strong electron transfer process, which is essentially the main reason behind the fact that porphyrins have good conversion between light and chemical/electrical energy (Werner et al., 2010). The porphyrins units can incorporate a metal atom through a chemical reaction usually termed as metallation, and the coordination chemistry of the metal centre can be used to introduce additional components and features to the overall assembly of porphyrin motifs as the organic dyes of choice for PV applications. The most common porphyrin systems studied for DSSCs are free-base and zinc derivatives of the *meso*-benzoic acid substituted porphyrin TCPP [tetrakis(4-carboxyphenyl)porphyrin]. These have an appropriate LUMO level that resides above the conduction band of TiO_2_ and a HOMO level that lies below the redox couple in the electrolyte solution, required for charge separation at the surface of the solar cell (Campbell et al., 2004). All above properties render porphyrin ligands as promising candidates in organic solar cells which allow them to act as “donor” molecule in DSSCs.

There are three main kinds of light-harvesting dye molecules and their recorded DSSC efficiency. The ruthenium-based sensitisers, which were first developed as dye molecules, show high power conversion efficiency, reported at around 10–11%. However, due to the toxicity and availability in low abundance on Earth, the ruthenium-based sensitisers are not suited for large scale fabrication and are not deemed environmentally friendly. The efficiencies of metal-free organic dyes were reported to be 9–10% during the recent 5 years, whereby the best-performed organic dye is C219, reaching *η* = 10.3% (Zeng et al., 2010). But, the trend in the performance progress of metal-free organic sensitisers seems to reach a bottleneck for their further development and there was no breakthrough discovery following on once direction has been proposed.

Table 4 shows some of the porphyrin molecules that have been applied and the cell power conversion efficiency. It can be seen that recent porphyrin-based DSSC development shows a promising advance with the progress curve. Porphyrin molecule applied as dye molecule in DSSCs was pioneering as reported by Kay and Grätzel; a mesoporphyrin IX dye was used and achieved a 2.6% (Kay and Graetzel, 1993). From then, there was no breaking research released, until 2004 when Md. K. Nazeeruddin reported that, by using a zinc centre porphyrin, corresponding to Zn-1a in Table 4, the DSSC efficiency can rise up to 4.6% (Nazeeruddin et al., 2004). Followed by their work, by applying Zn-3, Q. Wang and coworkers reported DSSCs with a 5.6% efficiency (Wang et al., 2005a). In 2007, the same group reported porphyrin sensitisers in another series, in which porphyrin GD2 in Table 4 exhibits a 7.1% efficiency (Campbell et al., 2007). As reaching 7.1% efficiency, porphyrins work as dye molecules in DSSCs could compete with organic small molecule even ruthenium sensitisers and open a great opportunity to enhance the efficiency by modifying their structure. In 2009, Kim and coworkers reported a zinc porphyrin (td-2b-bd-Zn) with a diarylamino group and reached an efficiency of 7.5% (Park et al., 2008). More excitingly, Yeh and Diau reported YD2 porphyrin with two long alkyl chains to improve the thermal and photochemical stability and obtain 11% efficiency (Bessho et al., 2010). Grätzel and coworkers reported an optimised performance DSSCs by using YD2-o-C8 and a cosensitised organic dye Y123 (Dualeh et al., 2011; Tsao et al., 2011); this cell efficiency can be up to 12.3%; the reason for this promising improvement is due to the upward shift of the TiO_2_ conduction band and the enhanced electron lifetime (Yella et al., 2011). The long alkyl chains play an essential role in diminishing the degree of porphyrin dye molecule aggregation.

**TABLE 4:** Porphyrin molecules used in some DSSCs which obtain high efficiency.

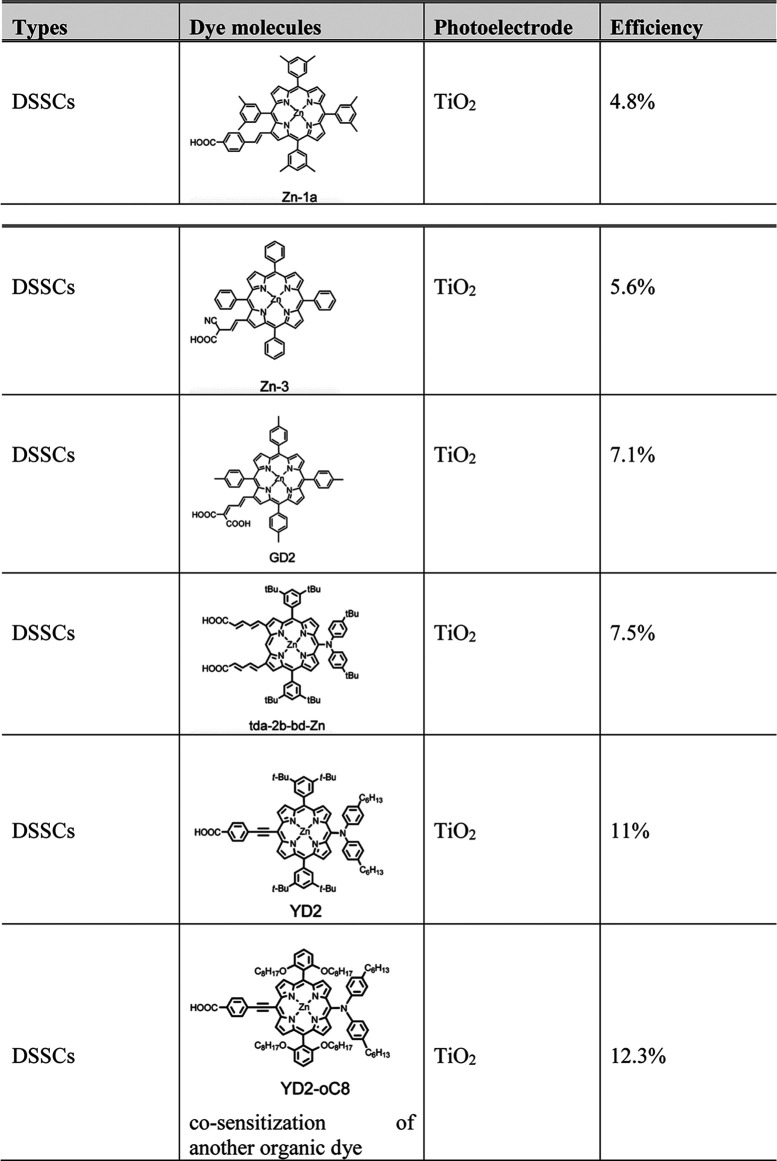

As described above, the development of cells efficiency goes alone with the porphyrin molecule modification. A very obvious modification method for the porphyrin molecule is to convert the free-base porphyrin into metalloporphyrin. Both experimental results and computer simulations have demonstrated that, by adding a meter centre, the absorption behaviours of porphyrin can be varied (Flamigni et al., 2000; Shubina et al., 2007). Zinc porphyrin molecules were the most common and widely used dye molecule in DSSCs due to the low cost and being nontoxic (D’Souza et al., 2001). Alongside metallation of the porphyrin core, the substitution of groups on the porphyrin can also allow tuning of the absorption the wavelengths, tailing of molar absorption coefficient, as a result of the HOMO/LUMO energy level modifications (Baerends et al., 2002; Zhang et al., 2005). As a result, grafting different groups around porphyrin ring have become one of the main pathways to improve electrochemical properties of porphyrins and thus improve the efficiency of DSSCs.

Functionalization and modification of porphyrin ligands have been carried out to overcome the problems presented by unfunctionalized porphyrins, which have previously been used as dye molecules in DSSCs. Porphyrins are highly conjugated and planar systems, which means that porphyrin has an inherent tendency to self-aggregate in solution at high concentrations. As stated above, the dipole/dipole interactions allow a rapid migration of the excited state between neighbouring dye molecules and this could lead to the annihilation of excitons, which in turn dramatically decrease the dye efficiency. Thus, the modification of the porphyrin ligand should not allow a high level of aggregation to occur. To date, it is generally accepted that the laboratory scale synthesis of the porphyrins is more challenging than that of most other dye molecules under investigation for DSSCs. The main reason is still due to the high aggregation trends but also due to purification issues, as the solubility in the most organic solvent is similar for the starting materials, intermediates, and final product. The flexibility in the synthesis of metalloporphyrins is introduced as much cheaper metals such as iron and zinc can be incorporated, which then contributes to the tuneable absorption wavelengths due to the nature of the π-electron network (Campbell et al., 2004). By altering the functional groups around the porphyrin, it is possible to alter the π-electron density on the ligand plane, which is then beneficial to the interactions of porphyrins and its versatility in engaging in donor-acceptor interactions is supramolecular system formation. Therefore, the strategy of modifying porphyrin molecules by introducing new functional groups around porphyrin was of interest recently (Wang et al., 2011). Porphyrin-based hybrid systems are able to avoid self-aggregation and capable of altering electron density and the injection pathway is of interest in DSSCs design.

As shown in Figure 8, there are two types of positions in the porphyrin molecule that can be used for molecule modification: the four *meso*-positions and eight *β*-positions. By introducing the functional group at the *β*-position, the resulting system can have an enhanced capability of electronic coupling of the dye with the surface of TiO_2_. This concept, to design *β*-functionalised porphyrin sensitisers, was initially explored by Kim and coworkers (Park et al., 2008). They demonstrated that zinc (II) porphyrin (tda-2b-bd-Zn, shown in Table 4) with two equivalent π-conjugated malonic-acid linkers effectively enhanced the efficiency of electron injection and retarded the charge recombination. Whilst the substituted linkers at the four *meso*-positions of the porphyrin have been used to suppress dye aggregation, the porphyrin YD2 (presented as Table 4) represented an attractive example of a *meso*-position modification. The functional groups introduced can be either highly conjugated groups or long alkyl chains. The long alkyl chains in porphyrins play an important role in diminishing effectively the degree of dye aggregation, which is crucial for an improved device performance since they generate interspace in the extended networks (Ripolles-Sanchis et al., 2012). Modifications of the porphyrin core through either the *meso*-position or the *β*-position linkage can lead to efficiency improvements of dye molecule towards DSSCs applications. Regardless of precise positions and choice of functional groups, the role of those modifications needs to overcome the problem of self-aggregation and enhance the electron injection properties when employing porphyrins as dye molecules in DSSCs.

We, and others, focussed on developing new and modified, functionalised porphyrins including of the groups at both the *meso*-position and *β*-positions (e.g., conjugated groups, and long alkyl chains) on a laboratory scale aiming to improve understand the challenges in the use of these dye molecules in PVs assembly (Mao et al., 2016; Mao et al., 2017; Mao et al., 2019). Substitution of the groups on the porphyrin molecule allows tuning of the adsorption wavelength, molar absorption coefficient, and the HOMO/LUMO levels (Seo et al., 2012). Various studies have shown that chemical substitution of porphyrin derivatives alters the donating efficiency in the DSSCs on the photovoltaic properties, due in part to either metallation modifying the transfer pathway from the porphyrin (Suzuki et al., 2011; Cooling et al., 2012) or the ability of electron-donating or withdrawing groups to increase or lower respectively the level of the (HOMO) of the porphyrin molecule (Archer and Nozik, 2008). Porphyrins with aromatic rings fused to the β-positions of the pyrrole residues are referred to as π-extended porphyrins, with the increased conjugation afforded by the fused rings to the porphyrin macrocycle leading to enhanced light absorption and efficient emission in the near-infrared (near-IR) region of the spectrum (Sommer et al., 2011).

Despite the advantages of using porphyrins as dyes in solar cells, there are still two main problems associated with porphyrin as components of a DSSC. The first is that, due to the highly conjugated and planar systems, porphyrins have an inherent tendency to self-aggregate at high concentrations unless bulky substituents are incorporated to separate the planes. Secondly, dipole/dipole interactions in aggregations allow rapid migration of the excited state between neighbouring dyes, increasing the probability of exciton annihilation (Campbell et al., 2004). It is therefore important to design systems that do not allow a high amount of aggregation to occur as this will reduce the cell efficiency. There are methods to overcome this problem such as protecting the porphyrin with an alkane thiolate. Here, the molecules still exhibit high light-harvesting capabilities whilst suppressing undesirable energy transfer quenching of the porphyrin singlet excited state (Hasobe et al., 2005).

The synthesis of porphyrins is considered more challenging than that of other dyes such as the original ruthenium-based dyes, the main reason being due to the same aggregation which prevents efficient separation during synthesis. However, it is the flexibility in the synthesis of porphyrins, as well as the ability to use much cheaper metals such as iron and zinc rather than ruthenium, which leads to the important properties of the dye (such as the mentioned tuneable absorption wavelengths and varied π-electron systems). Firstly, by altering the functional groups around the porphyrin, it is possible to alter the π-electronic density. This can be beneficial to the rational design of porphyrin-based supramolecular systems, for example, the separation of aromatic compounds via aromatic interactions (such as selective extraction of higher fullerenes) and in the design and synthesis of new materials with optoelectronic properties (Tong et al., 2011). It has also been demonstrated that there may be an optimum sensitiser orientation (or distance from surface) and linker length (or conjugation) dependence, wherein interfacial charge separation is still efficient but the charge recombination is inhibited (Campbell et al., 2004). Altering the structure of the porphyrin allows the tuning of the way it interacts with the surface of the cell, in both distance and orientation. Energy conversion efficiencies as high as 11% have recently been achieved, with a donor-acceptor substituted porphyrin sensitiser and an organic solvent-based electrolyte, thus making this family of dyes attractive for commercial application in DSSCs. The advantages of porphyrin sensitisers include reduced fabrication cost, a simple synthetic pathway, and a larger molar extinction coefficient compared to ruthenium-based sensitisers (Armel et al., 2011).

### Supramolecular Self-Assembly Processes of Relevance to DSSCs

Self-assembly in supramolecular chemistry is concerned with the spontaneous association of molecular components resulting in the generation of either discrete oligomolecular supermolecules or extended polymolecular assemblies (Beer et al., 1999). These multicomponent entities owe their existence to reversible interactions and so may dissociate and reform in response to particular chemical or environmental changes. The aggregation of these components gives rise to new entities with different properties that often behave in entirely novel and unexpected ways. The entropic loss in the generation of highly organised arrays in supramolecules is outweighed by the overall enthalpic gain (Beer et al., 1999).

Self-assembly may also be defined as the process by which a supramolecular species forms spontaneously from its components (Darcy et al., 2001). Namely, the total intermolecular interaction will rarely be greater than around 100 kJ mol^−1^ (whilst the weakest covalent bonds are on the order of 150 kJ mol^−1^) (Darcy et al., 2001).

The most famous example of supramolecular self-assembly is the double-helical form of DNA found in nature. Acidic hydrogen atoms donate to both oxygen and nitrogen accepting atoms. In synthetic chemistry, however, a range of intramolecular forces are employed including ion-ion interactions, ion-dipole interactions, dipole-dipole interactions, hydrogen bonding, interactions involving pi-systems, van der Waals forces, close packing forces, and hydrophobic effects.

**FIGURE 9:**
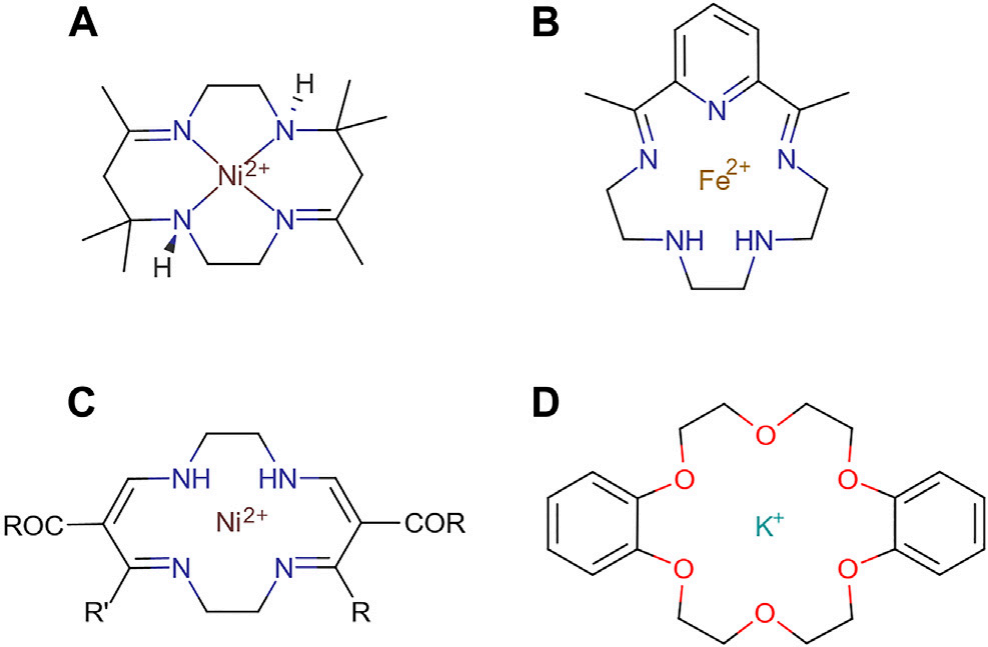
Schematic representations of the pioneering examples of molecules forming the building blocks of systems with supramolecular interactions reported by **(A)** Curtis, **(B)** Busch, **(C)** Jäger, and **(D)** Pederson.

The first reported example of a supramolecular molecule, one where two or more molecules are interacting via intramolecular forces, was given by Neil F. Curtis and coworkers in 1961 (Curtis and House, 1961), comprising a Schiff’s base macrocycle from acetone and ethylene diamine. Further examples of supramolecular interaction based on this metal-ligand coordination were given by Busch and Jäger in 1964 and Pederson in 1967 with the structures shown in Figure 9 (Steed and Atwood, 2009).

Host systems can be divided into several categories. Those with predominantly electrostatic forces are considered complexes. Those with nondirectional, less specific forces are split into two further categories: *cavitands* which contain intramolecular cavities, i.e., a “hole” in the molecule, and *clathrands* which feature extramolecular cavities (Vögtle et al., 1985). The driving force for the formation of these cavitand and clathrand supramolecular systems is the gain in entropy from the thermodynamically more favourable dense packing within the crystal structures (Cramer, 1956). Perhaps the best way to appreciate the applicability of this noncovalent approach in directing the synthesis of supramolecules is the one-step construction of nanometre-sized molecules using dynamic metal-ligand interactions. In most cases, gentle heating is required to reach thermodynamic equilibrium and the final products (from simple cages, bowls, boxes, capsules, and spheres to other high-symmetry three-dimensional architectures resembling Platonic and Archimedean solids) with extremely high kinetic stability can be obtained in high yields (Stang and Olenyuk, 1997).

Fullerenes, a class of π-carbon molecules such as C_60_ and C_70_ forming ball structures, are spontaneously attracted to porphyrins and metalloporphyrins. This particular type of supramolecular recognition was first discovered with cocrystallates of C_60_ and C_70_ with tetraarylporphyrins (Sun et al., 1997) and octaethylmetalloporphyrins (Hoeben et al., 2005). The unexpectedly strong interaction between a curved π surface and a flat π surface (C_fullerene_-to-porphyrin plane distance ∼2.7 Å) is largely van der Waals in origin (Boyd et al., 1999). It is still not possible to reliably predict the type of supramolecular structure that may form in the crystal in a given case, due to the competitive nature of the weak, noncovalent interactions involved and because the crystal formation is severely affected by subtle differences in the crystallisation environment (reflected often in pseudopolymorphism) (Diskin-Posner et al., 2002).

The combination of porphyrin as an electron donor and fullerene as an electron acceptor seems to be a promising candidate because of the following considerations:

1) The high light-harvesting efficiency of porphyrin throughout the solar spectrum.
2) Supramolecular complexation between porphyrin and fullerene due to π-π interactions (Imahori et al., 1996).
3) The efficient production of a long-lived, highly energetic charge-separated state by photoinduced electron transfer (ET) due to the small reorganisation energy involved in the ET (Imahori et al., 1996).

To further give light to these interactions, a macrocyclic extended tetrathiafulvalene (exTTF) host efficiently incorporates C_60_ or C_70_ with binding constants that range from 4.2 × 10^4^ to 3.8 × 10^6^ M^−1^. The binding is driven in large by charge-transfer interactions (Grimm et al., 2011). To date, three main subcategories of interactions of π-systems, D-H…π, π…..π, and cation….π, were receiving significant attention in the literature. The π-π interaction (stacking ∼0–50 kJ mol^−1^) is a nondirectional, electrostatic attractive force, which occurs when the attraction between π-electrons and π-framework overcome the unfavourable π- π repulsions. This interaction gives rise mostly to typical geometries such as edge to face (herringbone pattern) and offset face to face. Interestingly, it has been reported that a direct face-to-face geometry leads to a repulsive interaction.

Other features such as the polarisation of π-systems by heteroatoms may lead to direct face-to-face geometry. Such interactions are of significance both in nature (e.g., DNA structure) and in artificial systems, but are difficult to predict and control (due to their weak directionality and strength). The cation….. π interactions (5–80 kJ mol^−1^) occur between metallic or organic cations and aromatic or double/triple bonded regions of the molecule. These are based on electrostatic forces but relate also to the polarizability of the aromatics and have been associated with ion-induced dipole, donor-acceptor, charge-transfer, or dispersion forces interactions.

### Porphyrin Host-Guest Chemistry

Key components of the porphyrin (acting as a “host”) contribute to the supramolecular interaction through π-π interactions (from the extended aromatic π ring). Free-base porphyrins are capable of D-H-π interactions and metal porphyrins can form cation-π interactions to the neighbouring aromatic systems. Extensively conjugated π-frameworks such as these give rise to rich electrochemical and photophysical properties. The valuable spectroscopic properties of porphyrins (UV; NMR) help monitor reaction progress and also facilitate the characterisation of the final product. Moreover, the insertion of various metal ions into the porphyrin centre offers almost infinite possibilities for coordination and photophysical chemistry (Smith et al., 2002).

Broadly divided into two main categories, the assembling process can be achieved through axial coordination or bridging through external metal centres. Because of their photochemical and biomimetic properties and also the relatively large association constant (∼10^3^ M^−1^ in chlorinated solvents), porphyrin assemblies relying on the Zn-N (pyridine or imidazole) interactions are well-documented (Sessler et al., 1995).

### Porphyrin-Fullerene Blends

Porphyrin-fullerene supramolecular or covalently linked systems are considered to be very interesting classes of compounds due to their rich photo- and redox chemistry, remarkable photoactive, structural, and magnetic properties (Vijayaraghavan et al., 2012). Fullerenes present extraordinary electron-accepting characteristics, promoting ultrafast charge separation and exhibiting very slow charge recombination characteristics due to the low reorganisation energies involved. This in turn leads to the generation of a long-lived charge-separated state and a high quantum yield (D’Souza and Ito, 2009). The curved π surface of C_60_ shows a tendency to interact with other molecules, making it an interesting target for engineering supramolecular arrays (Boyd et al., 1999). Hoffman in 1995 (Eichhorn et al., 1995), looking for charge-transfer molecular crystals, obtained the crystal structure of octakis(dimethylamino)porphyrazine with C_60_ in a three-dimensional network where each C_60_ is sandwiched between two porphyrazines. With these crystals grown from toluene solution, they determined a distance from the centre of the porphyrazine to the centroid of the fullerene of 6.3 Å and they established the existence of van der Waals contact between them. As such there are numerous examples of combinations of porphyrins and fullerenes in the literature, both covalently (D’Souza et al., 2001; Imahori and Fukuzumi, 2004; Cho et al., 2005; Lehtivuori et al., 2006; Schuster et al., 2006; Umeyama and Imahori, 2006; Mathew et al., 2008; Iehl et al., 2011; Charvet et al., 2012; Tolkki et al., 2012) and noncovalently bound (Boyd et al., 1999; Konarev et al., 2002; Hasobe et al., 2005; D’Souza and Ito, 2009; Fathalla et al., 2009; Konarev et al., 2009; Nobukuni et al., 2009; Bhyrappa and Karunanithi, 2010; Hasobe, 2010; Oku et al., 2010; Wessendorf et al., 2010; Iehl et al., 2011; Kahnt et al., 2011; Sprafke et al., 2011; Wang et al., 2011; Konarev et al., 2012).

In 1997, Boyd & Reed (Sun et al., 1997) investigated if monosubstituted fullerides (C_60_^1-^) retained much of its parent C_60_ character. They prepared a pyrrolidine-linked tetraphenylporphyrin/C_60_ dyad and found that, in the solid stat, it packs forming self-assembled dimers. In two distinct crystallographic contacts, intradimer and interdimer, the distance between the closest C_60_ carbon atoms to the mean plane of the 16-atom inner core of the porphyrin ring is very short: 2.78 Å (interdimer) and 2.79 Å (intradimer). Graphite and other typical arene-arene separations are in the range of 3.3–3.5 Å, porphyrin-porphyrin separations are >3.2 Å (Hunter, 1994), fullerene-arene separations lie in the range 3.0–3.5 Å (Balch and Olmstead, 1999), and fullerene-fullerene separations are typically ca. 3.2 Å. This observation suggested that the π-π interaction between C_60_ and the porphyrin unit is substantially augmented probably due to strong donor-acceptor interactions. In contrast to the usual view of C_60_ as an acceptor, the sense of this donor-acceptor relationship might be with C_60_ acting as a donor and the porphyrin unit being the acceptor. If this was the case, one could expect that the more electron-rich bonds of the fullerene (6:6 junctures) should preferentially interact with the porphyrin unit.

In 1999, Boyd & Reed (Boyd et al., 1999) showed that the association of C_60_/C_70_ and tetraphenylporphyrins (TPPs) is also found in untethered cocrystallates and that this type of interaction also exists in solution. The TPPs used in the study possess aliphatic substituents and favourable “solvation” CH-π interactions of these residues with C_60_ and C_70_ were observed in the solid structures. This seems to be the next most important type of interaction in these systems after π-π interactions and is consistent with the relative solubility of C_60_ in arenes versus alkenes (Ruoff et al., 1993).

Ito et al. (Nojiri et al., 1998) in 1998 described an ET process that occurs when one of the two partners, the fullerene or the Zn porphyrin, is photoexcited and encounters the other in solution. It is a bimolecular process controlled by diffusion and it is possible if the concentration of the two species is high (they work around 10^–4^ M). The transient absorption bands for highly conjugated molecules such as C_60_/C_70_ are supposed to appear in the near-IR region of the spectrum. When the C_60_/C_70_ chromophore was predominantly photoexcited, ET took place from the ZnTPP ground state to the lower energy triplet excited state of the fullerene, 3C_60_*/3C_70_*, yielding the corresponding fulleride (C_60_·-/C_70_·-). On the contrary, when the species predominantly excited was the ZnTPP unit, it is the triplet state 3ZnTPP* that donates the electron to the ground state of the fullerene producing the corresponding anion radical C_60_·-/C_70_·-. The efficiency of the ET via photoexcitation to the triplet state of the fullerene is higher than the route involving the ZnTPP triplet state. This work highlights the potential of these systems as electronic materials.

In 1999, Diederich et al. (Armaroli et al., 1999) reported the binding of ZnTPP with a methanofullerene derivative bearing a pyridyl moiety. The photophysical properties of the assembly were studied and compared to the analogous fullerene monomalonate without the pyridyl group, which did not show any sign of complexation with the ZnTPP at μM concentration. The association constant value determined for the 1:1 complex, 3•ZnTPP, using ^1^H NMR titrations in C_6_D_6_ solution was K_a_ = 3.6 × 10^3^ M^−1^. This value is in good agreement with the one derived from the luminescence experiment in toluene, K_a_ = 3.0 × 10^3^ M^−1^.

The addition of large amounts of fullerene to the solution of ZnTPP produced a 50% diminution of the porphyrin fluorescence. Moreover, the absorption spectrum of the mixture showed a 10 nm redshift in the Soret band with respect to the free ZnTPP. A similar redshift is characteristic for axial binding of pyridine ligands to zinc porphyrins. This was the first noncovalent assembly, using coordination chemistry, reported in solution between fullerene and a porphyrin.

The fluorescence quenching of porphyrin in the presence of fullerene was attributed to a fast photoinduced energy transfer to the fullerene unit. Nevertheless, this statement is simply based on the reported preference of ET to occur in toluene when the porphyrin and the fullerene moieties are facing each other, whilst energy transfer happens when the components are located at longer distances (Kuciauskas et al., 1996). Additional proof favouring this hypothesis was derived from the known fast energy transfer mechanism reported through noncovalent bonds (Armaroli et al., 1997).

The reasons for this porphyrin-fullerene compatibility have been attributed to many factors. Among these are compatible shapes leading to numerous van der Waals contacts (Armaroli et al., 1997), π−π interactions between the fullerene surface and the porphyrin plane (Sun et al., 2000), charge-transfer interactions between the metal ions, such as Fe(II) or Co(II), and the fullerene acting as an electron acceptor (Sutton et al., 2004) or between the metal ion, e.g., Fe(III) and a fullerene acting as an electron donor (Evans et al., 1999), whilst dispersive (Liu and Schneider, 2002) and electrostatic interactions (Wang and Lin, 2003) were also considered. A recent review by Boyd and Reed (2005) summarises the complexity of the porphyrin-fullerene interactions and concludes that is “essentially van der Waals in nature but is perturbed by weak electrostatic and possible charge-transfer effects.”

**FIGURE 10:**
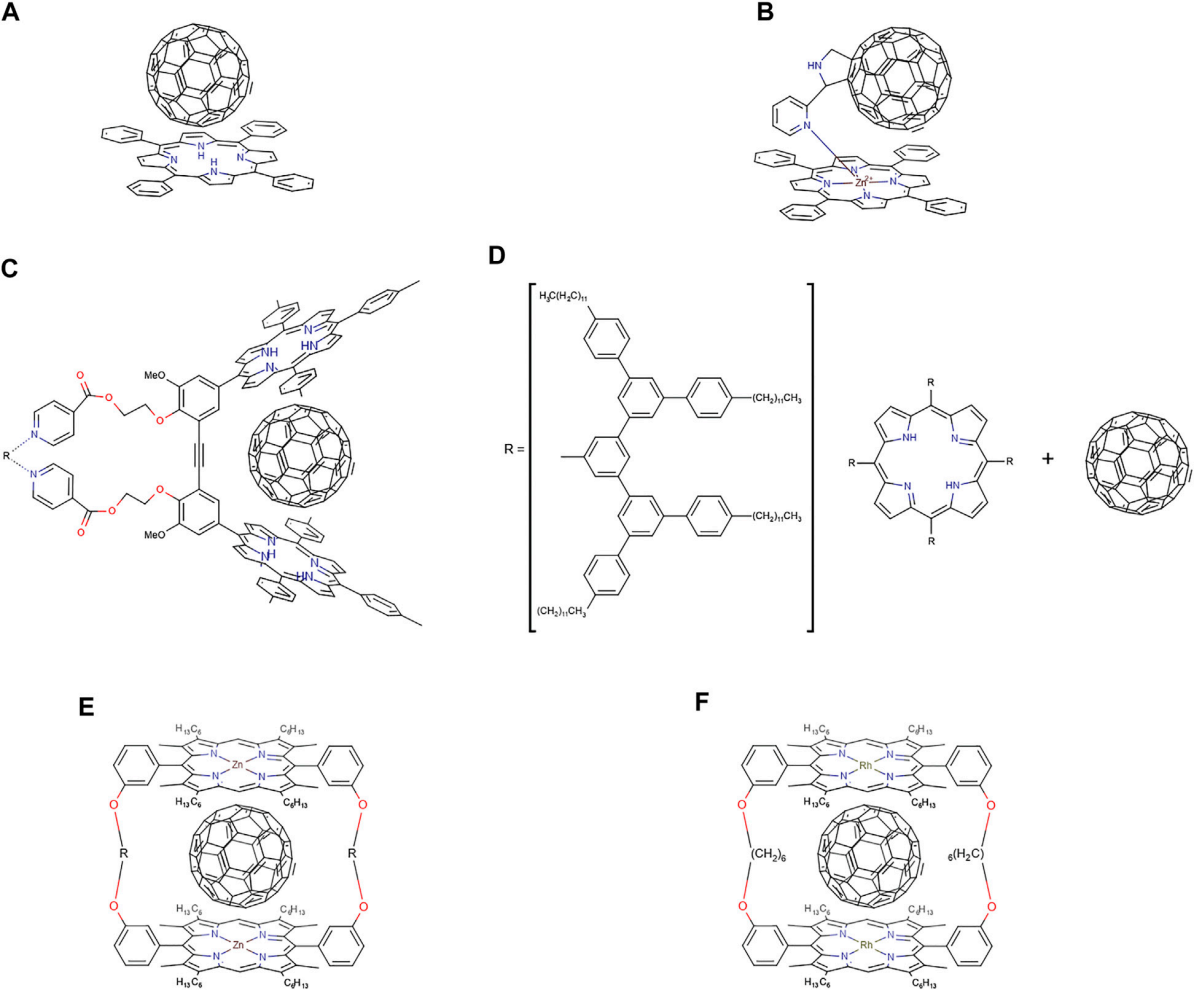
Representation of **(A)** cocrystallite-denoted H_2_TPP·C_60_ (Boyd et al., 1999), **(B)** zinc tetraphenylporphyrin C_60_ conjugate system (K_a_ = 7.3 × 10^3^ M^−1^) (Solladié et al., 2003), **(C)** supramolecular oligophenylenevinylene-C_60_ conjugate (K_a_ = 5.1 × 10^3^ M^−1^) (Gutiérrez-Nava et al., 2003), **(D)** components of a self-organised supramolecular complex composed of rigid dendritic porphyrin and C_60_ (K_a_ = 2.57 × 10^4^ M^−1^) (Kimura et al., 2002), **(E)** cyclic dimer of metalloporphyrin in a highly stable inclusion complex with C_60_ (K_a_ = 6.7 × 10^5^ M^−1^) R = (CH_2_)_6_ (Tashiro et al., 1999), and **(F)** cyclic rhodium (III) metalloporphyrin structure acting as host for fullerene molecules (K_a_, C_60_ = 2.4 × 10^7^ M^−1^; K_a_, C_70_ = 1.0 × 10^8^ M^−1^) (Zheng et al., 2001).

Figure 10 shows a selected range of supramolecular complexes of porphyrin-fullerene complexes which demonstrate the variety of interactions between the two molecules. Where known, the association constant (Ka) has been included to highlight the strength of these interactions.

**FIGURE 11:**
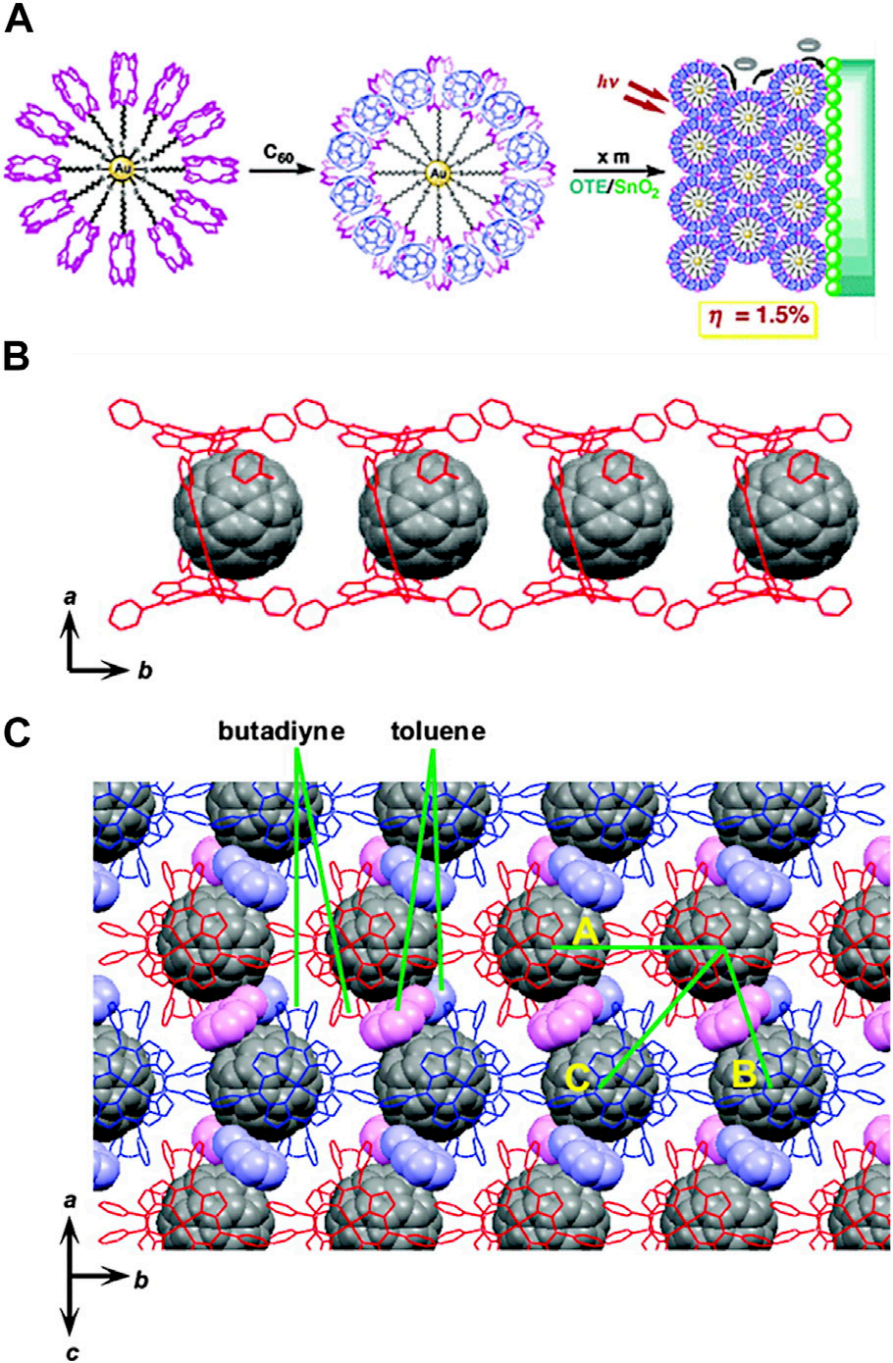
**(A)** Composite nanoclusters of porphyrins covalently bound to gold nanoparticles with fullerenes as acceptor molecules in between each porphyrin. Figure taken from Kamat et al. (Hasobe et al., 2005), crystal structures of tubular assemblies of a C_60_⊂Ni_2_−CPDPy donor-acceptor complex. Hydrogen atoms are omitted for clarity: **(B)** side view; **(C)** top view (Reprinted (adapted) with permission from Nobukuni et al., 2009. Copyright 2009 American Chemical Society).

In addition to supramolecular architects, covalent interactions allow the orientation and distances between the donors and acceptors to be designed prior to synthesis. Usually, highly conjugated connectors are used to allow for an efficient ET; however, this disrupts the π-electron system of the fullerene. Noncovalent binding and hydrogen bonding allow for the charge transfer without disruption of the π-system, as well as requiring no energy input to form the complex. This also means that the nanomaterials have scope for recycling as the two can be separated simply in solution by competitive solvation. However, the binding is understandably a lot weaker with noncovalent systems as it is limited to π-π interactions. Noncovalent binding however allows for some more creative systems, examples of which are shown in Figure 11.

A study by Kamat et al. demonstrated the importance of the metal centre within the porphyrin. They proved that the driving force of charge separation between excited zinc porphyrin and C_60_ is larger than a free-base porphyrin because the one-electron reduction potential of the singlet excited state of ZnP (−1.0 V vs. normal hydrogen electrode (NHE)) is more negative than that of H_2_P. Similarly, the shape of the acceptor fullerene molecule was also important, where replacing C_60_ with C_70_ as an electron acceptor in the composite electrode system may have different steric effects on the accommodation of C_70_ between two porphyrin rings as compared to C_60_ (Hasobe et al., 2005).

As mentioned before, one of the major reductions in efficiency arises from the recombination of charge carriers subsequent to absorption of a photon. Interface properties determine the electronic energy alignment in donor/acceptor interfaces and play an important role in controlling how the charge is separated and therefore preventing recombination (Kahnt et al., 2011). For example, a key issue in the electronic structure of organic interfaces is the donor-acceptor energy level alignment (Sai et al., 2012).

It is clear that this optimised orientation and energy level alignment are the most crucial elements to highly efficient light-harvesting properties. In some cases the stacking of porphyrin and fullerene molecules lead to interactions between the continuous domains of each, allowing for charge transfer in an oligomer-like manner (Wang et al., 2011). It is also possible for the porphyrins to form a single plane upon which the fullerene can then be deposited which can facilitate the efficient charge transport in heterojunction based solar cell devices (Zhong et al., 2012). A DFT study by S. Vijayaraghavan et al. showed that not only can the porphyrin molecule change its shape to a bowl-like structure to accommodate the fullerene molecule, but also the fullerene existed in three separate orientations, each with a unique level of conductance (Vijayaraghavan et al., 2012).

The interactions of porphyrins and fullerenes are still proving to be a widely researched topic, with applications in bulk heterojunction solar cells being the predominant application. There is still a wide range of porphyrins to synthesise, as well as many aspects of the interactions to solve meaning that this field has a lot of scope for further study.

### Carbon Nanotubes and Porphyrin Composites

Single-walled carbon nanotubes (SWCNTs) have recently become the focus of intense multidisciplinary study due to their unique structure, high mechanical strength and good chemical stability. They have been explored for several applications, ranging from solar cells to drug delivery, with each application requiring some modification of the nanotube. For their application in solar cells, SWCNTs can be seen as an expansion of a fullerene and thus fullerenes can be used to predict the interactions of porphyrins and nanotubes.

**FIGURE 12:**
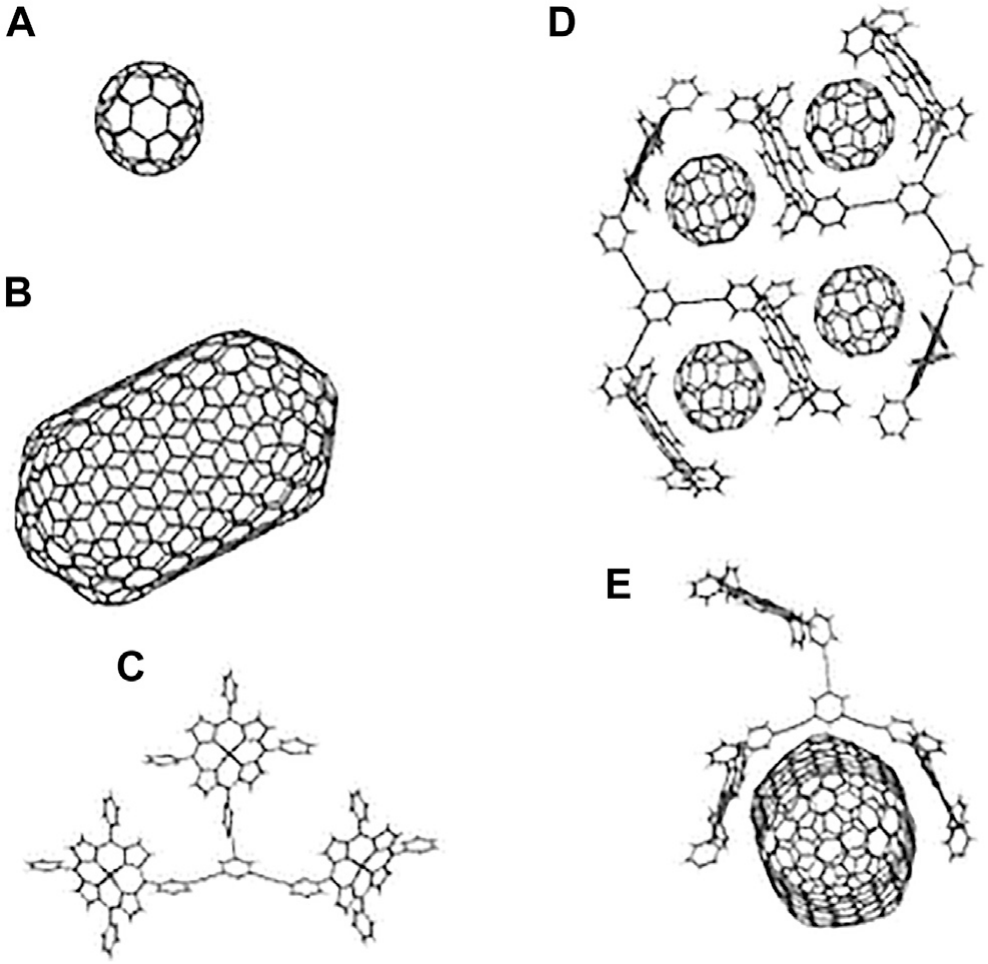
DFT-level optimised structures for the hosts and guests studied: **(A)** C_60_ molecule; **(B)** [10,10] capped SWNT; **(C)** simplified tripodal porphyrin host 1 (ZnP); **(D)** a 4 : 2 C_60_: porphyrin (ZnP) host complex; **(E)** porphyrin host (ZnP) [10,10] capped SWNT composite. Reproduced with permission from Pascu et al. (2008).

The advantage of using nanotubes is that not only is there the potential for several donor molecules per acceptor, but also due to the ballistic movement of electrons through the nanotube, the charge carriers can move through the nanotube from one donor to another almost at the speed of light (Mathew et al., 2008). Much like the noncovalent fullerene composites, supramolecular functionalisation of single-walled carbon nanotubes (SWNTs) has become a field of growing interest as this does not alter the sp^2^-bonded nanotube walls, thus maintaining the physical properties of the carbon nanotubes (Pascu et al., 2008). Noncovalent complexation is also an important method for improving the solubility of nanotubes and for introducing functionality, whilst not damaging the electronic structure. Composites based on this concept are being developed for sensors, field-effect transistors, hydrogen generation, and biomedical applications (D’Souza and Ito, 2009; Sprafke et al., 2011) (Figure 12).

### Graphene and Related 2D Carbon Nanomaterials in Porphyrin-Based Composites

A graphene sheet is essentially a dissected SWCNT and preserves the merit as a good electron acceptor due to its low reduction potential but does not suffer from the drawback of carbon nanotubes that could be either metallic or semiconducting (Zhang and Xi, 2011). Additionally, transparent, conductive, graphene electrodes for DSSCs have been reported, thus highlighting another potential utility of graphene in future nanotechnological applications (Karousis et al., 2011).

**FIGURE 13:**
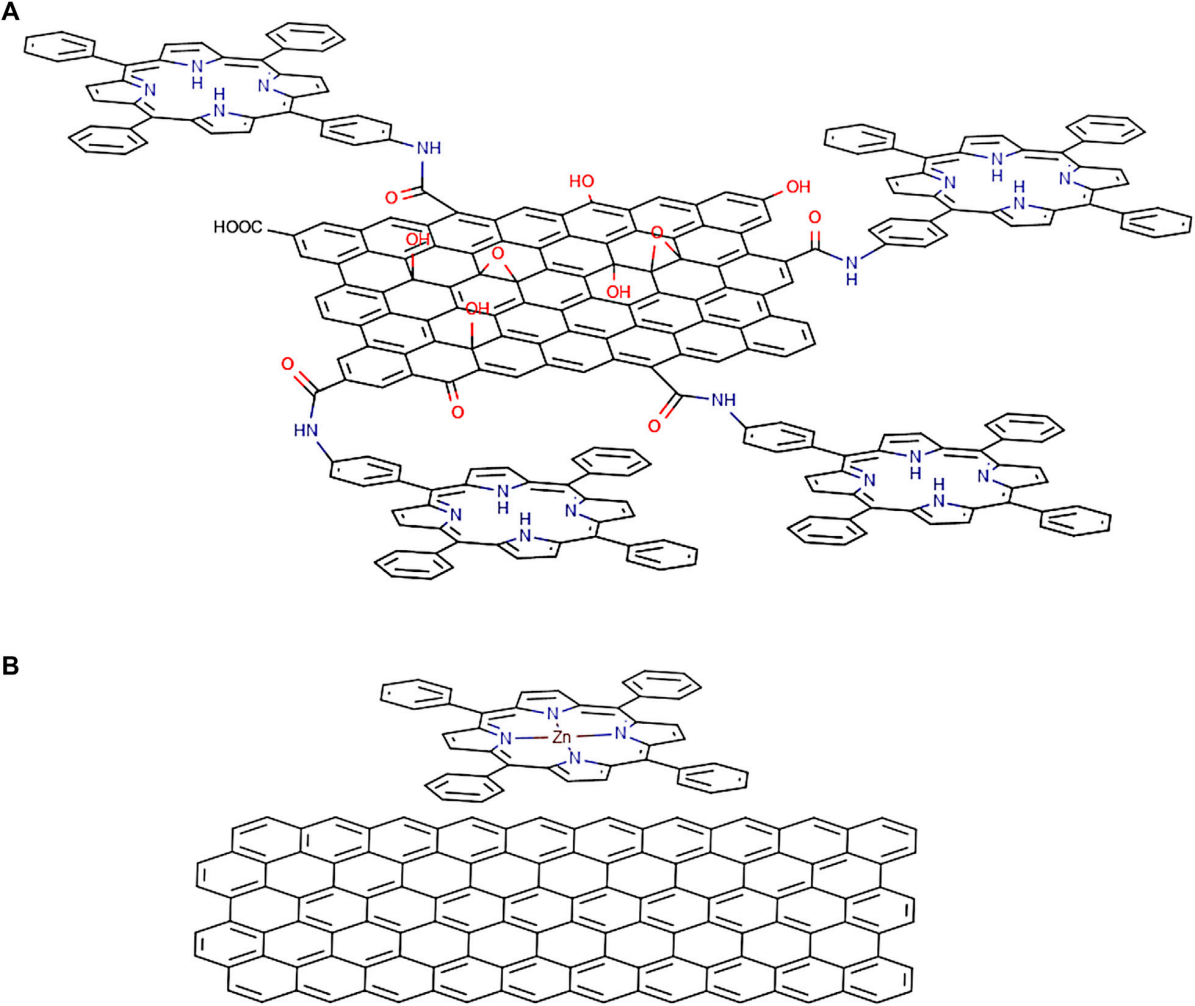
**(A)** Graphene oxide with covalently linked porphyrins (5-(4-aminophenyl)-10,15,20-triphenyl-21,23*H*-porphyrin) (Karousis et al., 2011); **(B)** the binding of a tetrasulfonated zinc phthalocyanine to graphene via π- π stacking (Zhang and Xi, 2011).

Ongoing research work to investigate porphyrin-carbon nanomaterial interactions is aimed at advancing the understanding towards improved porphyrin-based nanohybrids (Figure 13). The development of donor-acceptor based materials for solar cell applications is hampered by the limited availability of functional porphyrins accessible mainly on an analytical scale (Tong et al., 2011). Their laboratory scale synthesis and purification still represented significant challenges and the full understanding of their interactions with fullerenes and carbon nanomaterials represented uncharted territory.

Porphyrin cores used as either zinc(II) substituted or free-base centres as well as the incorporating peripheral substituents are meant to allow the fine-tuning of the electron density in the π-system. The inclusion of lengthy organic groups as the design elements was used as they were deemed necessary not only to help to “wrap” around carbon support (fullerene or carbon nanotube) to increase the binding strength but also to help to prevent self-aggregation due to aromatic stacking.

Approaches for functional porphyrins evolved from that initially designed for analytical work to a scalable process and is now amenable to laboratory scale synthetic routes (milligrams scale and beyond). Synthesis of functional porphyrins incorporating metal ions and/or lengthy organic chains as peripheral substituents was found to offer the additional practical benefit in that the separation of the final product is much simpler than that of standard porphyrin compounds reported to date, which do not incorporate long hexyl chains.

### Interactions Between Within Porphyrin Molecules and Carbon Nanomaterials

**FIGURE 14:**
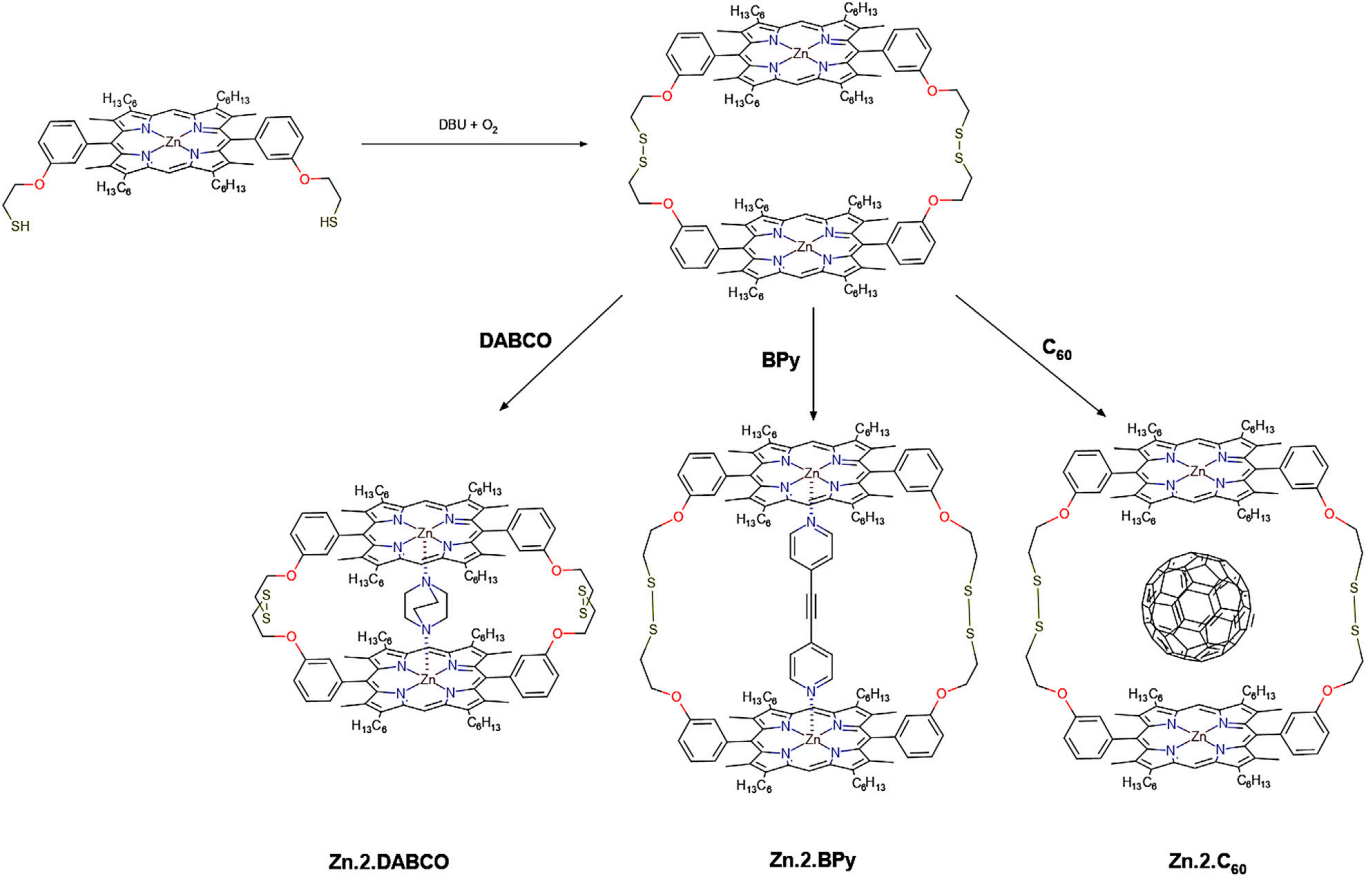
Binding of guests inside the adjustable porphyrin dimer receptors (Kieran et al., 2005).

As stated above, fullerenes are suitable systems acting as electron acceptor materials of relevance to organic solar cells, due to their capability of acting as highly efficient ET reduction media (Hunter, 1994). In some types of organic solar cells, the efficient induced ET occurs at the interface between the donor molecule and the fullerene layer interface. Whilst porphyrins can be regarded as one of the most efficient dye molecules in DSSCs due to present remarkable light-harvesting ability, as discussed above, their application in the presence of fullerenes, giving rise to donor and acceptor complex systems, seems to be ideal as an approach in solar cells applications. Figure 14 shows an example of the fullerene-porphyrin complex and its supramolecular assembly.

Porphyrins and fullerenes are known to form supramolecular complexes, which contain close contacts between one of the electron-rich module led ring bonds of the guest fullerene acting as a “donor” and the molecule of the host porphyrin acting as a “host” (Sun et al., 1997; Boyd et al., 1999; Sun et al., 2000; Sun et al., 2002a; Sun et al., 2002b). The contact distances in porphyrins and C_60_ are of the order of 2.7–3.0 Å, which is much longer than normal metal-olefin bonding distances commonly found in organometallic complexes (Balch and Olmstead, 1999). The porphyrin-fullerene interaction energies are reported to be in the range from −16 to −18 kcal mol^−1^ (Wang and Lin, 2003), which is a very strong force capable of driving the formation of porphyrin and C_60_ supramolecular complexes. The interactions between porphyrin and fullerene can be explained by π-π interaction involving van der Waals forces. But, it is worth noticing that, in a study by Reed, a free-base porphyrin was found to bind to a fullerene exhibiting a more strong link than metalloporphyrins and a suggested explanation was due to the importance of electrostatic interactions involved. The noncovalently binding and hydrogen bonding allow the charge transfer without disruption of the π-system. Also, no additional input energy is required at the formation of such hybrids. In some cases, the metal centre in the metalloporphyrin can be linked with C_60_ in a combination with metal-ligand coordination interaction. An example is shown in Figure 14 and this linking strategy also provides a novel noncovalently bonding type in fullerene-porphyrin complex design.

The functionalised Zn(II)-porphyrins of interest for this project have been isolated on an analytical scale for dynamic combinational chemistry studies. Dynamic combinatorial chemistry methods were also applied to generate new compounds held together by fullerene and porphyrin donor-accepter interactions. Dynamic combinatorial chemistry relies on molecular recognition and is a new approach to synthesise molecules and their complexes on an analytical scale (Corbett et al., 2006). Dynamic combinatorial libraries were carried out by linking simple building blocks together with a reversible reaction under thermodynamic control. The libraries are allowing a constant interchange of building blocks resulting in a mixture of library members in equilibrium (Corbett et al., 2006). In the fullerene and porphyrin system, the dynamic combinational concept was applied by regarding a porphyrin dimer as a receptor and fullerene as a guest. Amy L. Kieran reported several approaches in porphyrin and fullerene dynamic combinatorial chemistry, but the limitation of that work was the tiny scale on which the new compounds were isolated (Kieran et al., 2005; Kieran et al., 2005).

The combination of fullerene and porphyrins can also be addressed by covalently linking such molecules (Drovetskaya et al., 1995; D’Souza et al., 2002; de la Torre et al., 2005). The covalent binding or the donor and the acceptor allow the orientation and distances between porphyrin and fullerene to be designed in advance. In the covalently linked complexes, highly conjugated linking groups are employed to allow for an efficient ET, but this change can alter the π-electron system in both the porphyrin and the fullerene systems.

It has been shown that complexes incorporating a fullerene and a porphyrin show great promise as synthetic scaffolds for new functional materials for photovoltaic applications but due to the synthetic challenges so far, only a few investigations into the device fabrication based on porphyrin oligomers and carbon nanotubes or graphene/graphene oxide could be carried out (Murakami et al., 2003a). It is known that, from the class of carbon-based nanomaterials, carbon nanotubes and graphene/graphene oxides also have promising electronic and mechanical properties in their own right, which makes them even more interesting when combined with porphyrin molecules as the resulting nanohybrids can act as a donor and acceptor system. Nakashima first reported that a complex formed by porphyrin and single-walled carbon nanotubes via noncovalent interactions (Murakami et al., 2003a). After this study, some research has been carried out in this area: Raghu Chitta reported zinc (II)-porphyrin noncovalently linked to single-walled carbon nanotubes and showed that these syntheses are held together by π-π interactions (Chitta et al., 2007). The covalent linking strategies were investigated to an even more limited extent: Zhen Guo reported a method to covalently link porphyrin with SWNTs by a diazonium group (Guo et al., 2006). Similar reactions were carried out between graphene/graphene oxides with a porphyrin substrate. Nikolaos Karousis reported a covalently linked porphyrin with graphene by diazonium group, as a direct adaptable converting from Zhen Guo’s research (Karousis et al., 2012). In 2012, Murali Krishna reported a covalently linked porphyrin and graphene oxide with metal or metal-free porphyrin by using the defects group on the edge of graphene oxide (Krishna et al., 2012).

New modes of assembling between carbon nanomaterials and porphyrins are being investigated. These showed great promise as new synthetic scaffolds for functional materials for photovoltaic applications (Tong et al., 2008). Our new nanohybrids have been designed to incorporate with SWNTs and graphene/graphene oxide and explore by both supramolecular self-assembly method (which typically leads to the π-system of the carbon nanomaterials to remain unaltered) and a more synthetically demanding route, living a covalent approach to link the donor and accepter (but disruptive for the experimental aromatic synthesis) (Tong et al., 2008).

## Carbon Nanomaterials

As one of the most abundant elements on the planet, carbon is the *materia prima* for life and the basis of all organic chemistry. Other than the naturally formed carbon allotropes, human-made carbon allotropes also presented promising properties. Fullerenes, regarded as zero-dimensional materials are the molecules where carbon atoms are arranged spherically and carbon atoms in this molecule fill the sp^2^ orbital (Andreoni, 2000). Recently, Andrey Chuvilin et al. directly visualised a process of fullerene formation from a graphene sheet using aberration-corrected transmission electron microscopy (Chuvilin et al., 2010a). Carbon nanotubes can be regarded by rolling graphene sheet along a certain direction and then relinking the carbon bonds. Thus, carbon nanotubes can vary in length and be thought of as one-dimensional material. The discovery of carbon nanotubes was converted from C_60_. The synthesis of multiwalled carbon nanotubes (MWNTs) was firstly reported by Iijima in 1991 (Iijima, 1991), as a result of an experiment where he pursued the synthesis of fullerenes via the arc discharge method. After further study, single-walled carbon nanotubes (SWNTs) were then reported in 1993 (Iijima and Ichihashi, 1993), and a method was specifically designed to produce such higher fullerenes by adding transition-metal catalysts (for example, Ni and Y) to the graphite in an arc discharge method. Since then carbon nanotubes have been extensively considered for applications in many different research fields, due to their structural, physical, and electronic properties such as high chemical and thermal stability, high elasticity, tensile strength, metallic conductivity, and large surface area (Terrones, 2003). SWNTs have diameters 0.5–5.0 nm (commonly around 1–2 nm), whereas the diameter of MWNTs is 2–100 nm (commonly around 10 nm). The lengths of carbon nanotubes are typically in the micrometres range, but in some reports, it has been shown that these can be up to millimetres or even centimetres (Zhu et al., 2002). The inner space of carbon nanotubes, which are encapsulated, is employed as a nanoreactor, to monitor or control the reactivity of fullerene and other small molecules to form some unusual supramolecular and covalent structures (Khlobystov, 2011).

### Functionalisation of Carbon Nanotubes

The functionalisation of carbon nanotubes has already become a very important research field. As some functional groups are grafted on the surface or trapped within the inner space of carbon nanotubes, those functional groups can alter the properties of carbon nanotubes, such as increasing the SWNTs solubility, exfoliate and monodisperse bundles of the tube, include higher-order structures using self-assembly, and so on. Although the functionalisation of SWNTs has attracted some significant attention, the chemistry of functionalised carbon nanotubes is very challenging due to the poor solubility and low reactivity of the SWNTs. The functionalised results could be very hard to characterise due to batch-to-batch recording in SWNTs starting materials available.

The strategies of functionalisation of carbon nanotubes could be divided into five types: 1) surface defect-group functionalisation, 2) covalent sidewall functionalisation, 3) noncovalent stacking functionalisation with surfactants, 4) noncovalent stacking functionalisation with polymers, and 5) endohedral functionalisation with nanoparticles. The covalent functionalised strategies include surface defect-group functionalisation and covalent sidewall functionalisation. The oxygen-containing surface defects can be introduced onto the surface of SWNTs by oxidation reactions (Zhang et al., 2003). It has been reported that the use of highly reactive reagents to directly attack the side wall is another way to functionalise carbon nanotubes (Strano et al., 2003). The use of reactive agents and methods, borrowed and adapted from C_60_ chemistry, such as the Bingel (Coleman et al., 2003) reaction and radical trapping reaction (Liu et al., 2006) have been particularly successful.

It has been found that the noncovalent functionalisation strategies for carbon nanotubes can avoid the introduction of defects into the carbon nanotubes structure. The noncovalently functionalisation methods are based on surface association and van der Waals Interactions (Rance et al., 2010). For example, a lot of effort has been devoted to study the interaction between long chains of DNA and nanotubes (Meng et al., 2007; Tu et al., 2009). It is believed that the nanotubes are in the hydrophobic interiors of the corresponding micelles, which results in stable dispersions. When the hydrophobic part of the amphiphile contains an aromatic group, an especially strong interaction results, because of an effective and cooperative, extended π-π stacking interactions which can then be formed with the graphitic sidewalls of the SWNTs. This effect was demonstrated in the aggregation of CNTs with N-succinimidyl-1-pyrenebutanoate (Chen et al., 2001).

For the endohedral functionalisation strategy, C_60_ molecules have also been found to be encapsulated into the inner space of nanotube to form a hybrid C_60_@SWNTs structure (Okada et al., 2001; del CarmenáGimenez-Lopez, 2011). Filling nanotubes with metal nanoparticles have been also successful in both original growth methods (Demoncy et al., 1998) and liquid phase filling methods (Hsin et al., 2001; del Carmen Giménez-López et al., 2011). Research reporting the encapsulation of metallofullerene has also been published (Chuvilin et al., 2010b; Zhang et al., 2011; Chamberlain et al., 2011a; Maggini et al., 2014). It is also reported that graphene nanoribbons are easier self-assembly together inside the inner space of carbon nanotubes (Chuvilin et al., 2011). Since carbon nanotubes are highly heterogeneous, many functionalisation techniques have been used in an attempt to separate single-wall nanotubes samples, which include dielectrophoresis, selective functionalisation, wrapping with DNA and density gradient centrifugation (Hersam, 2008). All those methods are limited to small volume and low yield, but once the single-walled carbon nanotubes are separated, the unique diameter and chirality of SWNTs could be studied, and it was shown that, depending on diameters and lengths, such SWNTs can be highly coloured (Tu et al., 2009).

### Carbon Nanotubes as Synthetic Scaffolds for Photovoltaics

Carbon nanotubes have a variety of attractive features which make them a suitable material for electrochemistry: high electrical conductivity; natural hydrophobic and adjustable porosity. One application for which these have been widely studied results in the use of carbon nanotubes in electrochemistry, and the application of carbon nanotubes as substrates is considered for the improvement of traditional solar cell electrode designs. In addition, the carbon nanotube can absorb light in both UV and near-IR range. It has been investigated that the use of SWNTs can add benefits both in terms of performance and durability of PVs (Du Pasquier et al., 2005; Rowell et al., 2006).

For organic bulk heterojunction solar cells, the diffusion ranges of excitons remained a problem since the diffusion can only occur in a couple of nanometres near the interface between donors and acceptors. In this case, one of the improvements of OSCs is the introduction of mixed donor and acceptor species and provides the device with a much larger contact interface between donors and acceptors. The diffusion range of excitons in an organic photovoltaic device is approximately in the order of 10 nm. When mixing the donors materials and carbon-based nanomaterials acceptor materials together, the actual size of the donor layer and that of the acceptor are smaller than the diffusion range and the domain can be reduced to several nanometres. Thus, the excitons can diffuse to the interface much easier and quicker. SWNTs can potentially be applied as the acceptor material in this improvement, due to their unique properties, like low electrical resistance and large surface area. Until recently, followed by this principle, some studies reported that SWNTs have been introduced in OSCs as the acceptor materials (Cataldo et al., 2012), Luyao Lu and coworkers have demonstrated a method to use N-doped multiwall carbon nanotubes as acceptor materials which led to the achievement of highly efficient polymer bulk heterojunction solar cells (Lu et al., 2013). It has also been found that, in DSSCs, the electron injection from the excited state of the dye molecule occurs on timescales much faster than the excited state decay and recombination of the injected electron with the dye cation (Haque et al., 1998; Tachibana et al., 2002). Thus, similar to BHJ, it has been found that when single-walled nanotubes were used as the material of choice for providing solar cells with necessary electrochemical properties, the resulting solar cells have been significantly improved in terms of efficiency (Kongkanand et al., 2007; Brown et al., 2008; Yan et al., 2013). Therefore, it is always demonstrated that SWNTs can play an important role in improving the charge separation, and it was shown that the injected electrons for conduction become faster in the presence of the SWNT scaffold (Dembele et al., 2013). Some recent researches also showed that when a quantum dot-single-wall carbon nanotube complex is used as an additive, the resulting solar cell shows a good performance of light covert efficacy (Landi et al., 2005).

### Introduction of Graphene Oxide and Graphene as Materials of Choice for PV Applications

Graphene rapidly became one of the most popular research areas since it was discovered by A. K. Geim and K. S. Novoselov at the University of Manchester, by using a deceptively simple Scotch tape method (Geim and Novoselov, 2007). The reason behind this rapid development of this material is due to its promising properties: graphene has a significant large surface area, 2,630 m^2^ g^−1^, which is nearly hundreds times larger than normal carbon material; it has high intrinsic mobility, 200,000 cm^2^ V^−1^ s^−1^; graphene also obtains high Young’s modulus, around 1.0 TPa; the thermal conductivity of graphene is 5,000 W m^−1^ K^−1^ and its optical transmittance is up to 97.7%. Besides these physical characteristics, graphene sheets have a special electronic structure, giving this material remarkable electronic properties, for instance, the anomalous quantum Hall effect (Novoselov et al., 2007) and its amazingly high carrier mobility at a relatively high charge carrier concentration and at room temperature (Bolotin et al., 2008). All these properties rendered graphene as a new material considered to be in next-generation applications of several different types of nanoelectronic devices of relevance to sensing and biosensing, transparent conductive films construction, fuel cells, electrical energy storage, and also structural materials design. The lattice of a monolayer of graphene sheet lattice consists of two equivalent sublattices of carbon atoms bonded together with σ bonds. The delocalised network of electrons within a graphene sheet is due to each carbon atom in the graphene lattice obtaining a π orbital.

There are normally three strategies to produce graphene: 1) exfoliation of graphite in solvents, 2) micromechanical exfoliation, and 3) epitaxial graphene. The production of graphene by micromechanical exfoliation was introduced by Ruoff and coworkers (Lu et al., 1999). Although the micromechanical exfoliated graphene has high quality and leads to the rapid development of graphene characterisation, this method is not suitable for larger material production scales and thus cannot meet the requirements for most commercial applications. The direct production of graphene by exfoliating graphite in common organic solvents, such as DMF and NMP, was introduced, and this included a prolonged time under the ultrasound treatment (Hernandez et al., 2008). This direct conversion can produce graphene with low a defect ratio, but besides the fact that it is time- and energy-consuming, it is still limited by the large size and thickness of the sheets produced (Lotya et al., 2009). It is also reported that a high-quality thin graphene film could be produced from fast electrochemical exfoliation (Su et al., 2011). The epitaxial graphene is introduced by chemical vapour deposition (CVD) on metallic catalysts. This method has been shown to be used in the generation of a high-quality monolayer or several layers of graphene with very thin dimensions and which is highly conductive (Reina et al., 2008; Li et al., 2009; Losurdo et al., 2011). The epitaxial graphene production is currently the most widely studied method. The large scale production of graphene of high carbon purity by CVD approaches could provide the desired material in sufficient quantity for a host of likely applications, as well as for fundamental science. But, the challenge for epitaxial graphene production is the efficient transfer of the generated graphene sheets from the metal substrate to other substrates. Until recently, a new method for graphene generation is emerging, which is described as a substrate-free gas-phase synthesis of graphene (Dato et al., 2008). This method provides a new approach to the synthesis of graphene.

As discussed above, although graphene has several different promising properties, the large generation is still very challenging. In order to find a cost-effective method for the production of graphene, graphene oxide reemerged as an intense research area (Dreyer et al., 2010). The graphene oxide has similar structural properties compared with graphene. Graphene oxide can be regarded as a defective graphene sheet, which functionalised by introducing oxygen-containing groups (including carboxyl groups, hydroxyl groups, and carbonyl groups) onto its surface. The properties of graphene oxide change with the changing of the percentage of oxygen groups.

The generation of graphene oxide is via the direct exfoliation of graphite oxide. Graphite oxide has a similar structure to graphite, but the plane of carbon atoms in graphite oxide is heavily decorated with oxygen-containing groups, which not only enlarge the distance between each layer but also make the thin layer hydrophilic and thus they are increasing soluble in the aqueous phase. Thus, graphene oxide can be exfoliated from graphite oxide by moderate sonication. Generally, graphite oxide is synthesised by either Brodie (1860), Staudenmaier (1898) or Hummers method (Hummers and Offeman, 1958). Brodie and Staudenmaier used a combination of potassium chlorate (KClO_3_) with nitric acid (HNO_3_) to oxidise graphite, whilst the Hummers method involves the treatment of graphite with potassium permanganate (KMnO_4_) and sulfuric acid (H_2_SO_4_). The level of the oxidation can be varied on the basis of the method, the reaction conditions and the precursor graphite used.

After the introduction of oxygen-containing groups onto the surface of a graphene sheet, graphene oxide sheets are significantly hydrophilic and the water molecules can intercalate into the inner layer space (Buchsteiner et al., 2006). Thus, the graphene oxide can form a stable aqueous colloidal suspension. The inner distance between each graphene oxide sheet in a water colloidal suspension could vary from 6 to 12 Å, depending on the increasing humidity and oxidation, and this can make its characterisation particularly challenging (Buchsteiner et al., 2006). Recent studies showed that the water dispersed graphene oxide exhibits a negative surface charge (Li et al., 2008). The negative charge on the graphene oxide surface leads to electrostatic repulsion between negatively charged graphene oxide sheets and contribute to making the aqueous suspensions very stable. The negatively charged surface of graphene oxide in water dispersions is a very important property when applied to the role of graphene oxide in substrate deposition processes from dispersed phases as the surface charge of the substrate material should be matched (Li et al., 2008).

### Reduced Graphene Oxide

The reduction of graphene oxide was applied as one of the alternative ways to produce graphene in bulk generation. The reduction of graphene oxide can be carried out by several methods. Graphene oxide can be reduced by a high-temperature annealing process. The thermal reduced process can decompose the oxygen-containing groups and exfoliate graphene oxide sheet at the same time. According to Gao’s research, the critical dissociation temperature (Tc) of hydroxyl groups attached to the edges of GO is 650°C and only above this temperature hydroxyl groups can be fully removed. After the thermal annealing at temperatures of 700–1,200°C in a vacuum, the hydroxyl groups can be fully eliminated (Gao et al., 2009). The good reduction effect by thermal annealing at around 1,000°C was also proved by the high conductivity reported by Becerril et al. (2008) and Wang et al. (2008). As theory predicted, the common annealing reduction has to be carried out at 900–1,100°C, such that the oxygen groups on the surface of graphene oxide can be efficiently removed and the ratio of C/O may be significantly increased (Eda et al., 2008). As an alternative process, microwave irradiation reduction of graphene oxide has been carried out (Hassan et al., 2009; Zhu et al., 2010). The main advantage of microwave irradiation over other heating sources is that the heating of substances can be uniform and rapid. By treating graphite oxide powders in a commercial microwave oven, graphene oxide can be readily reduced to the product within 1 min.

Another type of graphene reduction is carried out by introducing chemical reducing agents. The chemical reduced graphene oxide can be obtained by processes carried out at room temperature or moderate heating atmosphere. The most common and widely used reducing reagent is hydrazine or hydrogen hydrate (Gómez-Navarro et al., 2007; Stankovich et al., 2007; Mattevi et al., 2009). The reduction by hydrazine and its derivatives, such as dimethylhydrazine (Stankovich et al., 2006), can be addressed by adding the liquid reagents to a GO aqueous dispersion. This results in the formation of agglomerated graphene-based nanosheets due to the increase of synthetic’s hydrophobicity. The use of NaBH_4_ for this process was also reported recently. This can be used as the reducing reagent to produce rGO (Shin et al., 2009). As discussed above, the use of thermally reducing and chemically reducing reagents are the most common methods applied in the reducing progress of graphene oxide. A novel method that combines thermal and chemical reducing methods together emerged recently: a hydrothermal process where overheated supercritical water can play the role of a reducing agent (Zhou et al., 2009b; Dubin et al., 2010).

Other methods, like photoreduction (Zhang et al., 2010), photocatalyst reduction (Williams et al., 2008), and electrochemical reduction (Zhou et al., 2009a), have all been reported for rGO production, used to reduce graphene oxide, compared with the thermal reduction and chemical reagent reduction methods; these methods are not suitable in bulk reduction of graphene, as the reducing rates are not very applicable for large scale applications.

### Graphene and Graphene Oxide as Material Scaffolds in Photovoltaic

Since the graphene sheets hold promising thermally conductive, electronic, and mechanical properties, graphene and its divertive/graphene oxide are considered as scaffolds towards the construction of next-generation flexible photovoltaic devices. It was reported by Xuan Wang and coworkers by employing graphene as a material for the electrode of DSSCs. As made, obtained graphene films exhibit a high conductivity of 550 S/cm and transparency of more than 70% over 1,000–3,000 nm (Wang et al., 2008). Gurpreet Singh Selopal reported that, by employing graphene as a transparent front contact layer material, a value of photoconversion efficiency as high as 2% has been recorded for the best cell, under one sun irradiation (Selopal et al., 2014).

Similarly, with the care of SWNTs, the graphene and graphene oxide can also be used as electron acceptors due to their low reduction potential. For graphene sheets, the electrons can freely move at room temperature. This significant electron transport ability renders graphene an ideal material to assist the charge separation and conduction in solar cells systems. Considering its size, a graphene sheet can measure up to the micrometre range, which is a thousand times larger than the common donor molecules used in DSSCs. As a result, graphene can provide abundant space for donor molecule to attach either covalently or noncovalently.

**FIGURE 15:**
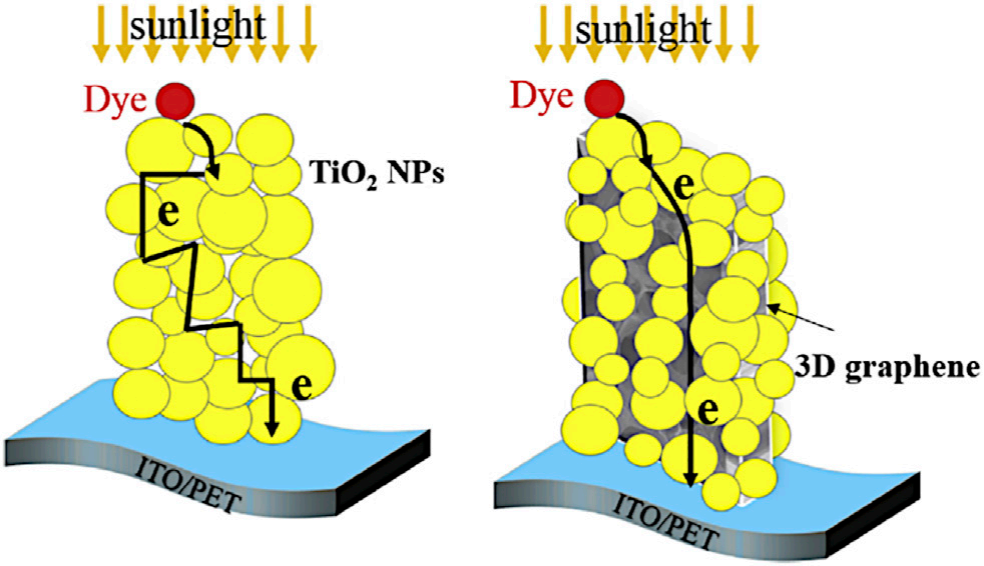
Schematic illustration of the devices and electron transport path without and with graphene (ITO-indium tin oxide; PET-polyethylene terephthalate) (Zhi et al., 2015).

Additionally, to the considerations discussed above, Jacob Tse-Wei Wang also reported an electron collection layer consisting of graphene-TiO_2_ nanocomposites as components of thin-film perovskite solar cells. This showed a remarkable photovoltaic performance with a power conversion efficiency of up to 15.6% (Wang et al., 2013). Recently, Jian Zhi demonstrated that graphene, TiO_2_, and a dye molecule complex anode structure (as showing in Figure 15) can achieve a power conversion efficiency of 6.41%. This is 56% higher than the care when the pristine TiO_2_ material was used for the anode (Zhi et al., 2015). This design confirmed that graphene can be used as an acceptor material in organic solar cells. Other researchers found that CdS and graphene can lead to a high photo degradation rate under visible light irradiation (Pan and Liu, 2012) and similar results have also been found for CdSe quantum dots with graphene oxide (Lightcap and Kamat, 2012). As such, although graphene and graphene oxide are new materials, they are already becoming of interest for solar cells and related PV devices consideration and study.

We and others focussed recently on new studies towards the synthesis of nanoassemblies porphyrins and carbon nanomaterials. These already showed some great promises as new synthetic scaffolds for functional materials for photovoltaic applications but have not yet been investigated in full (Tong et al., 2008). We reported recently on the lab scale preparation of new functional porphyrins modified in both *meso*- and *β*-position and metalled to obtain a more tuneable UV-vis absorption. Such functional porphyrins have been deemed challenging to synthesis on a lab scale previously; however, in our recent studies, we isolated these and studied their interactions with carbon nanomaterials (Mao et al., 2016; Tyson et al., 2016a; Mao et al., 2017; Calatayud et al., 2019; Mao et al., 2019; Owen et al., 2020).

The generation of new porphyrin-base complexes incorporating SWNTs as well as graphene oxides was achieved by both applying supramolecular self-assembly (relying on the π-system of the carbon nanomaterials, will remain unaltered) and a more synthetically demanding (and disruptive) covalent approach linking methods. We also reported recently on our investigations regarding the surface modification and functionalisation of SWNTs, synthesis of graphene oxide, and reduction of graphene oxide and performed the characterisation of new porphyrin-carbon nanomaterial blend by TEM, SEM, AFM methods, and Raman spectroscopy. New highly conjugated and soluble porphyrins with tunable UV-vis absorption have been developed by incorporating metal centers. Systematic characterization of their properties has been carried out by UV-vis, fluorescence spectroscopy and single photo confocal microscopy. In addition, the energy levels of the molecular orbitals of the gas-phase porphyrin system have been investigated by DFT calculations. This work will give the possibilities in exploiting porphyrin and carbon nanomaterials complex into real photovoltaic applications.

## Summary to Sustainable Energy

In summary, solar energy and related sustainable technologies should and could play more important roles in societal daily life in the near future. Organic solar cells exhibit great promise for the future of photovoltaics, due to their high versatility and accessibility among different types of solar cell fabrication techniques. For organic solar cells, small molecules can be employed as the light absorber; this has been demonstrated for both organic bulk heterojunction solar cells and for DSSCs. Although the cells discussed above all differ in structure, the basic operating principle of BHJs and DSSCs is largely the same: the light absorber works as a “donor,” which absorbs light and generates exited electrons; afterwards, the electrons are transferred to “acceptor” and then transferred to an external current.

It can be seen from the above discussions, either in the developments of bulk heterojunction solar cells or due to the recent revolutions leading the photovoltaics development from DSSCs to perovskite solar cells and from perovskite solar cells to further proposed developing directions, that almost all attempted efforts are based on synthesising and characterising new donor/accepter materials and improving the electron diffusion efficiency within the systems. Thus, this review highlighted current challenges and opportunities highlighted by a small molecule-based donor incorporating a porphyrin motif, which can lead to novel nanodimensional materials-based acceptor systems. These may be of relevance to future application in bulk heterojunction solar cells or DSSC structures.
